# Plant growth-promoting effects of a novel *Lelliottia* sp. JS-SCA-14 and comparative genome analysis

**DOI:** 10.3389/fpls.2024.1484616

**Published:** 2024-11-26

**Authors:** Byeong Jun Jeon, Jin-Soo Park, Sung-Chul Hong, Eun Ha Lee, Jaeyoung Choi, Jeong Do Kim

**Affiliations:** ^1^ Smart Farm Research Center, Korea Institute of Science and Technology, Gangneung, Republic of Korea; ^2^ Natural Product Systems Biology Center, Korea Institute of Science and Technology, Gangneung, Republic of Korea; ^3^ Department of Food Science and Biotechnology, Kunsan National University, Gunsan, Republic of Korea; ^4^ Department of Oriental Medicine Biotechnology, College of Life Sciences, Kyung Hee University, Yongin, Republic of Korea

**Keywords:** plant growth promotion, phosphorus solubilization, siderophore, biostimulant, *Lelliottia*

## Abstract

Bacteria associated with plants play crucial roles in promoting plant growth and health by aiding in nutrient acquisition, including phosphorus. This study presents the isolation and genomic characterization of a potentially new bacterial strain, *Lelliottia* sp. JS-SCA-14, which exhibits significant plant growth-promoting effects through phosphorus solubilization. A comparative phylogenomic analysis of the complete genome of strain JS-SCA-14 and its closely related strains revealed a unique genomic profile, suggesting it could be a novel species. Genomic identity calculations indicated that JS-SCA-14 significantly deviates from strains belonging to closely related genera, such as *Buttiauxella*, *Citrobacter*, *Enterobacter*, *Leclercia*, and *Lelliottia*. A biochemical assay comparing JS-SCA-14 and a closely related strain, *Lelliottia jeotgali* PFL01^T^, showed differing patterns in carbon source utilization and enzyme activities. To assess the plant growth-promoting capabilities of strain JS-SCA-14, tests were conducted to evaluate its siderophore-producing and phosphate-solubilizing abilities. Seed germination assays demonstrated an improvement in germination, seedling length, and vigor compared to untreated controls. Notably, the phosphate-dissolving strain JS-SCA-14 led to a significant increase of 34.4% in fresh weight and 35.4% in dry weight of tomato plants compared to the negative control. These findings underscore the significant potential of strain JS-SCA-14 in solubilizing phosphate, thereby enhancing phosphorus availability in the rhizosphere and promoting plant growth and development. This study contributes to our understanding of plant-microbe interactions and suggests the potential application of strain JS-SCA-14 as a bioinoculant for sustainable agriculture and plant nutrient management strategies.

## 1 Introduction

The *Lelliottia* genus, a Gram-negative, rod-shaped, motile, and facultative anaerobic bacterium, belongs to the *Enterobacteriaceae* family. In 2013, based on multilocus sequence type, DNA-DNA hybridization, phenotypic sugar fermentation characteristics, and cell wall fatty acid profile analysis, *Enterobacter amnigena* and *E. nimipressuralis* were proposed as a new genus derived from *Enterobacter*, named *Lelliottia* (Brady et al., 2013). Over the past few years, *Lelliottia* and close relatives in the family *Enterobacteriaceae* showing plant growth-promoting (PGP) have been also characterized. *Lelliottia amnigena* MSR-M49 has been shown to improve wheat growth by enabling it to tolerate salinity stress through the reduction of Na^+^ ions toxicity in saline soils (El-Akhdar et al., 2020). *Enterobacter* sp. and *Lelliottia* sp. treatments enhanced the growth of tomato roots and shoots during the early vegetative development stage (Kalozoumis et al., 2021). Two novel plant growth-promoting strains of *L. amnigena*, isolated from *Euphorbia prostrata* Aiton, have recently been reported to enhance the overall productivity of wheat and tomato (Parashar et al., 2023). This suggests that the *Lelliottia* genus could be more efficiently utilized to improve crop productivity.

Microorganisms that colonize the rhizosphere and promote plant growth are classified as plant growth-promoting rhizobacteria (PGPR), which are commonly found in soil. These free-living PGPR, identified as bacteria from various genera, show promise as biocontrol agents (Kloepper et al., 1989; Pahari et al., 2017). The direct effects of PGPR on plant growth are facilitated by the secretion of phytohormones, solubilization of inorganic phosphates, fixation of atmospheric nitrogen, increased iron nutrition through iron-chelating siderophores, and reduction of ethylene concentration (Glick et al., 1998; Hussain et al., 2013). Moreover, PGPR indirectly promotes growth by reducing the population of root pathogens and harmful microorganisms in the rhizosphere. This is achieved through the production of antibiotics and hydrolytic enzymes, the induction of induced systemic resistance and systemic acquired resistance, and competition for space and nutrients (Saharan and Nehra, 2011). Therefore, PGPR can serve as an alternative to chemical fertilizers, pesticides, and supplements. They also offer more efficient biological control strategies, thereby enhancing crop-growing systems.

Phosphorus (P) is a crucial macronutrient required for plant growth and energy metabolism. Despite the abundance of phosphorus in the soil, only a small proportion is available for plant uptake. Biological systems offered by microorganisms make insoluble inorganic P in the soil available to plants (Saharan and Nehra, 2011). Certain microorganisms play a vital role in increasing plant yield by converting insoluble P into soluble dihydrogen phosphate (H_2_PO_4_^–^) and hydrogen phosphate (HPO_4_^2–^) ions (Billah et al., 2019). Phosphate-solubilizing bacteria increase the availability of phosphates to plants, promoting plant growth and improving soil fertility.

Siderophores are iron-chelating agents secreted by microorganisms to solubilize iron in the soil. Given the essential requirement of ions for the growth and metabolism of nearly all living organisms, these compounds play a central role in symbiotic interactions with plants, benefiting both the plant and the bacterium (Crowley, 2006; Ghosh et al., 2020). Siderophores produced by PGPR can increase plant iron uptake and protect plants from pathogenic bacteria and fungi. Besides improving plant growth siderophores, enhance soil structure by chelating other micronutrients in the soil, making them available to plants, and promoting the growth of beneficial microorganisms (Deb and Tatung, 2024).

Consequently, microorganisms exhibiting dual traits of phosphate solubilization and siderophore production are considered a promising source of plant growth-promoting agents for sustainable agriculture. Hussain et al. (2019) reported that the interaction between phosphate-solubilizing bacteria such as *Pseudomonas*, *Mycobacterium*, *Bacillus*, *Pantoea*, *Rhizobia*, and *Burkholderia* with phosphate fertilizers improved wheat grain yield by 22% and phosphorus uptake by 26%, while reducing fertilizer application by 30% (from 120 kg P_2_O_5_ ha^-1^ to 90 kg P_2_O_5_ ha^-1^). These biofertilizers are safe and non-toxic to the environment. Thus, this study was designed to identify new PGPR strain that can be used as biofertilizers to improve tomato plant production. Efficiently identifying a bacterial strain with PGP traits that influence plant growth is possible using *in vitro* assay systems for potential PGPR screening (Singh et al., 2020). The screening system for selecting PGPR strains involves the solubilization of inorganic phosphorus available to plants, the production of plant hormone modulators such as ACC (1-aminocyclopropane-1-carboxylate) deaminase, the production of plant growth regulators like indole acetic acid (IAA), gibberellic acid (GA), and cytokinins (CKs), and the exhibition of antagonistic activity against plant pathogens by siderophore production. Four *in vitro* assay methods are used for the isolation and screening of rhizobacteria to select potential PGPR strains. The JS-SCA-14 strain, a novel species candidate, which showed phosphate solubilization and siderophore production ability *in vitro* was finally selected and evaluated for its ability to promote the growth of tomato plants. Additionally, the strain was studied to determine whether it constitutes a new species through further taxonomic analysis and genomic characteristic analysis.

## 2 Materials and methods

### 2.1 Isolation of soil bacteria

Bacterial strains were isolated from soil samples around plants inhabiting the sediment at Cheongnyeongpo, Yeongwol, Republic of Korea. Fifty g of soil sample (37°10'32" N 128°26'31" E) was collected at a depth of 10-15 cm using a soil sampler (GeoSampler 5350-5005, Burkle Inc., Germany). In the isolation process, 1 g of soil sample was mixed with 9 mL of sterile water in a 15 mL conical tube. The tube was then shaken at 28°C for 1 h on a rotary shaker at 200 rpm. Subsequently, the suspension was serially diluted and spread on a starch casein agar (SCA) medium. The SCA medium composition includes 10 g soluble starch, 0.3 g casein, 2 g KNO_3_, 0.05 g MgSO_4_·7H_2_O, 2 g K_2_HPO_4_, 2 g NaCl, 0.02 g CaCO_3_, 0.01 g FeSO_4_·7H_2_O, and 18 g agar in 1 L water. The plates were then incubated at 28°C for 2–3 days. Bacterial colonies with distinct morphologies on the plates were initially maintained by repeated streaking on fresh SCA plates. The isolated strains were subsequently utilized for PGPR screening.

### 2.2 Isolation of chromosomal DNA

JS-SCA-14 strain was grown on tryptic soy agar (TSA) at 28°C for 2 days. Genomic DNA extraction was carried out using the method described by Mehling et al. (1995). In summary, the cells (0.1–0.2 g) were resuspended in a microtube containing 500 µL TE buffer (25 mM Tris and 25 mM EDTA, pH 8.0), and then 2 µL of lysozyme (10 mg mL^-1^) and RNase A (50 mg mL^-1^) were added, followed by incubation at 37°C for 30 min. Subsequently, 50 µL of 5 M NaCl was added, and the suspension was vortexed and lysed by adding 120 µL of 10% SDS. The lysate was incubated for 30 min at 65°C. After adding 240 µL of 5 M potassium acetate, the solution was mixed by vortexing, placed on ice for 20 min, and centrifuged at 13,000 rpm for 5 min at 4°C. The supernatant was transferred to another tube. Phenol/chloroform/isoamyl alcohol (25:24:1) was added, vortexed for 30 s, and centrifuged at 13,000 rpm for 10 min at 4°C. The supernatant was transferred to another tube. Then, 0.6 volume of isopropanol was added, and after at least 5 min at 4°C, the precipitated DNA was pelleted by centrifugation in a microfuge. After a wash with 70% ethanol, the pellet was dried for 10 min and resuspended in 30 µL of distilled water.

### 2.3 Sequencing and genome processing

The genome of strain JS-SCA-14 was sequenced by combining PacBio Sequel and Illumina sequencing technologies (Macrogen, Inc., Seoul, Republic of Korea). PacBio and Illumina sequencing produced 138,991 and 11,024,576 (sub) reads with total base counts of 1,275,124,138 and 1,663,452,532, respectively. The reads were assembled using the Microbial Assembly application of PacBio SMRT analysis pipeline v8.0. The chromosome and plasmid sequences were further corrected using Illumina reads with Pilon v1.21 (Walker et al., 2014). The quality of the gene prediction was evaluated using Benchmarking Universal Single-Copy Orthologs (BUSCO v5.4.7) with enterobacterales_odb10 lineage data (Manni et al., 2021). Protein-coding and RNA genes were predicted using Prokka (v1.13) (Seemann, 2014) and RNAmmer (v1.2) (Lagesen et al., 2007), respectively. Genomic features, including predicted genes and genomic islands, were graphically presented using Circos (v0.69-9) (Krzywinski et al., 2009). The predicted genes were functionally annotated through the eggNOG-mapper (Cantalapiedra et al., 2021), which utilizes Clusters of Orthologous Groups (COGs) categories.

### 2.4 Phylogenetic and phylogenomic analysis for genus and species identification

The identification of the species was conducted based on the consensus sequence of the 16S rRNA genes (1,530 base pairs) predicted from the genome. This sequence was analyzed with the EZBioCloud 16S database (version 2023.06.29) (Yoon et al., 2017), and significant hits were extracted for further analysis. For the phylogenomic analysis, the DNA-DNA relatedness value was determined by calculating the digital DNA-DNA hybridization (dDDH) using the recommended settings of the genome-to-genome distance calculator (GGDC v3.0) (Meier-Kolthoff et al., 2013, 2022). In addition, average nucleotide identity values were computed using the orthologous average nucleotide identity tool (OrthoANI v1.40) (Lee et al., 2016). The dDDH and OrthoANI calculations were visually represented through a scatter plot by using the R programming language (v4.2.3) with the ggplot2, ggExtra, and ggthemes packages (Wickham, 2016; Arnold, 2021; R Core Team, 2021; Attali and Baker, 2022).

### 2.5 Bioinformatics analysis

The complete genome of strain JS-SCA-14 was annotated to identify biosynthetic gene clusters (BGCs) and plant growth-promoting traits (PGPTs) by using antiSMASH and PGPT-Pred, respectively (Blin et al., 2021; Patz et al., 2021). Genomic islands and putative horizontal gene transfer events were identified by using IslandViewer (v4) and Alien_Hunter (v1.7) (Vernikos and Parkhill, 2006; Bertelli et al., 2017). To illustrate the relationship between strain JS-SCA-14 and 957 closely related species within the genera *Buttiauxella*, *Citrobacter*, *Enterobacter*, *Leclercia*, and *Lelliottia* (**Supplementary Table 1**), a phylogenomic tree was constructed using the standalone version of Composition Vector Tree (CVTree) (Qi et al., 2004). The proteome sequence of *Escherichia coli* str. K-12 substr. MG1655 (GCF_000005845.2) served as an out-group for the phylogenomic tree. A *K*-tuple length of six was selected for CVTree, as it has been suggested as the optimal value for bacterial phylogeny (Zuo et al., 2010). The phylogenomic tree was visually presented, depicting the homology distribution of protein sequences using graphical phylogenetic analysis (GraPhlAn v1.1.4) (Asnicar et al., 2015).

### 2.6 Phenotypic and chemotaxonomic characterization

The strains JS-SCA-14 and *L. jeotgali* PFL01^T^ were grown in tryptic soy broth (TSB) for 2 days for subsequent characterization. Their morphological features were examined using a field emission scanning electron microscope (Regulus 8100, Hitachi, Japan). The API ZYM Biochemical Test Kit (bioMérieux, Marcy l**’**étoile, France) was employed to evaluate the biochemical characteristics in accordance with the manufacturer**’**s instructions. The ability of the strain to utilize a single carbon source was investigated on basal media supplemented with 1% of the carbon source (Shirling and Gottlieb, 1966). Cellular fatty acids were extracted, methylated, and then analyzed using a 6890N gas chromatography system (Agilent Technologies, Santa Clara, CA, USA) in conjunction with the Sherlock microbial identification software package (version 6.1) (Sasser, 1990).

### 2.7 Screening for bacteria with IAA production and ACC deaminase activity

Strains isolated from soil were inoculated into King’s B medium (per liter: 20 g proteose peptone No. 3, 1.5 g K_2_HPO_4_, 1.5 g MgSO_4_·7H_2_O, and 10 mL glycerol) supplemented with 5 mM L-tryptophan (Trp) and cultured in a rotary shaker for 2 days at 28°C and 200 rpm. The culture broth was centrifuged at 4°C and 8000 rpm for 5 min to collect the supernatant. Then culture supernatant and Salkowski reagent (27.6 mM FeCl_3_ and 6.6M H_2_SO_4_ dissolved in distilled water) were mixed at a ratio of 1:3, and incubated in the dark for 30 min. The absorbance was measured at 530 nm using a spectrophotometer to confirm the IAA production ability of strains isolated from soil. The standard curve was measured at 530 nm with IAA (Sigma-Aldrich, St.Louis, MO, USA) concentrations of 0, 5, 10, 20, 50, and 100 µg mL^-1^. The ACC deaminase activity of strains isolated from soil was evaluated as follows. Single colonies of each strain were streaked onto Dworkin and Foster (DF) salt minimal agar medium (per liter: 4 g KH_2_PO_4_, 6 g Na_2_HPO_4_, 0.2 g MgSO_4_·7H_2_O, 2 g glucose, 2 g gluconic acid, 2 g citric acid, and 15 g agar with trace elements: 1 mg FeSO_4_·7H_2_O, 10 µg H_3_BO_3_, 11.1 µg MnSO_4_·H_2_O, 124.6 µg ZnSO_4_·7H_2_O, 78.22 µg CuSO_4_·5H_2_O, and 10 µg MoO_3_; pH 7.2) as a negative control, DF salt minimal agar medium containing 2.0 g/L (NH_4_)_2_SO_4_ as a positive control and DF salt minimal agar medium containing 3 mM ACC as the sole nitrogen source instead of (NH_4_)_2_SO_4_ as an experimental group. and the inoculated plates were incubated at 28°C for 2 days. Strains that did not grow in the negative control group but grow in the experimental group at a level comparable to the positive control group were selected.

### 2.8 Screening for phosphate-solubilizing bacteria

The bacteria isolated were tested for their ability to solubilize phosphate on Pikovskaya (PVK) agar medium, which contains tricalcium phosphate (TCP) as a substrate (Pikovskaya, 1948). For the initial screening, a single colony grown on TSA medium was streaked onto PVK agar. For the secondary screening, the single colony was transferred to a 15 mL conical tube containing 5 mL of TSB and incubated at 28°C for 2 or 3 days on a rotary shaker at 200 rpm. The phosphate-solubilizing activity was evaluated by depositing 10 μL of the inoculum onto PVK agar and observing clear/halo zones after 7 days of inoculation. The phosphate-solubilizing activity was quantified by calculating the phosphate-solubilizing index (PSI) using the formula [colony diameter + halo zone diameter)/colony diameter] (Premono et al., 1996).

### 2.9 Screening for siderophore-producing bacteria

The ability of isolates to produce siderophores was tested on Chrome Azurol S (CAS) agar plates, which contain an iron-dye complex that changes color when iron is lost (Schwyn and Neilands, 1987). The CAS agar consisted of a blue dye solution, a mixture solution, and PIPES [piperazine-N,N’-bis (2-ethanesulfonic acid)]. The blue dye solution was prepared using three solutions: solution 1 (0.06 g CAS dissolved in 50 mL of distilled water), solution 2 (0.0027 g FeCl_3_·6H_2_O dissolved in 10 mL of 10 mM HCl), and solution 3 (0.073 g of hexadecyltrimethylammonium (HDTMA) dissolved in 40 mL of distilled water). The mixture solution was prepared with 100 mL of minimal media 9 (MM9) salt solution (per liter: 30 g KH_2_PO_4_, 50 g NaCl, and 100 g NH_4_Cl), 30 mL casamino acid solution (3 g casamino acid dissolved in 27 mL of distilled water extracted with 3% 8-hydroxyquinoline in chloroform to remove any trace iron), and 10 mL of 20% glucose solution. The PIPES solution (pH 6.8) was prepared by adding 100 mL of MM9 salt solution to 750 mL of ddH_2_O. The solution was autoclaved after adding 15 g of agar. The casamino acid solution and 20% glucose solution were added to the PIPES/MM9 mixture. The blue dye solution was added last, with sufficient agitation to ensure thorough mixing.

### 2.10 Seed germination

Tomato seeds (cv. Seogwang) were surface-sterilized with 0.02% sodium hypochlorite for 2 min and then rinsed three times with sterile distilled water. Strain JS-SCA-14 was cultivated in TSB at 28°C for 24 h on a rotary shaker at 200 rpm, after which it was diluted with sterile distilled water to a final concentration of 10^8^ cfu mL^-1^. Twenty-five tomato seeds were inoculated with the bacterial suspension and a sticker solution (2.5 g gum acacia + 5 g sugar in 100 ml distilled water) for 30 min prior to sowing. Seeds treated solely with the sticker solution served as the non-treated control. For each treatment, the seeds were arranged on filter paper in a Petri dish (15 cm diameter) containing 10 mL of sterile distilled water and incubated in a growth chamber at 28°C. After 7 days, the number of germinated seeds was counted. The root and shoot length of each seedling was measured using the method of Abdul-Baki and Anderson (1973), with the vigor index calculated as: Vigor index = (root length + shoot length) × % germination. The experiment was conducted 25 times independently, with three replicates for each treatment.

### 2.11 Pot experiment

The impact of selected PGPRs on the growth and development of tomato plants was studied through experiments conducted under greenhouse conditions. The JS-SCA-14 strain, known for its efficacy in solubilizing inorganic phosphate, was selected for the experiment. A modified Hoagland solution (114 mg NH_4_H_2_PO_4_, 606 mg KNO_3_, 944 mg Ca(NO_3_)_2_·4H_2_O, and 492 mg MgSO_4_·7H_2_O with trace elements: 1.54 mg H_3_BO_3_, 0.45 mg MnSO_2_·4H_2_O, 18.5 mg Fe-EDTA, 0.57 mg ZnSO_4_·7H_2_O, 0.12 mg CuSO_4_·5H_2_O, 0.02 mg Na_2_MoO_4_·2H_2_O, and 1L distilled water) (Hoagland and Arnon, 1950) was used as a nutrient solution for tomato plants. TCP served as the source of the insoluble phosphate. The experiment comprised five treatments: T1, where tomato plants were supplemented with water; T2, where tomato plants were supplemented with Hoagland solution without NH_4_H_2_PO_4_; T3, where tomato plants were supplemented with Hoagland solution; T4, where uninoculated tomato plants were supplemented with Hoagland solution containing TCP as a P-deficient solution; and T5, where JS-SCA-14-inoculated tomato plants were supplemented with Hoagland solution containing TCP as a P-deficient solution. Tomato seeds were washed as described above and pre-germinated at 28°C for 5 days. These pre-germinated seeds were sown in a 36-plug tray (25 × 25 × 6 cm) filled with perlite (20 g) as nutrient-free soil. The bacterial cell count was adjusted to 10^8^ cells mL^-1^ in a 10 mM MgSO_4_ buffer and then applied around the roots of the tomato plants once a week for 4 weeks. Tomato plants treated with the MgSO_4_ buffer served as the nontreated control. All solutions were added at 3-day intervals. The experimental units were arranged in a completely randomized design. The average temperature was maintained at 25  ± 2°C with a light-dark cycle of 14 h of light and 10 h of darkness. After 30 days, the fresh weight of the tomato plants was determined, and the dry weight was measured after drying the plants in an oven at 50°C for 3 days. The experiment was conducted six times for each treatment. Data (n = 6) were analyzed using one-way analysis of variance, and the significance of mean differences between treatments was determined using Tukey’s honest significant difference (Tukey HSD) test (*p* < 0.05) with R software version 4.2.3 (R Core Team, 2021).

## 3 Results and discussion

### 3.1 Phylogenetic and phylogenomic analyses for species identification

**Figure 1:**
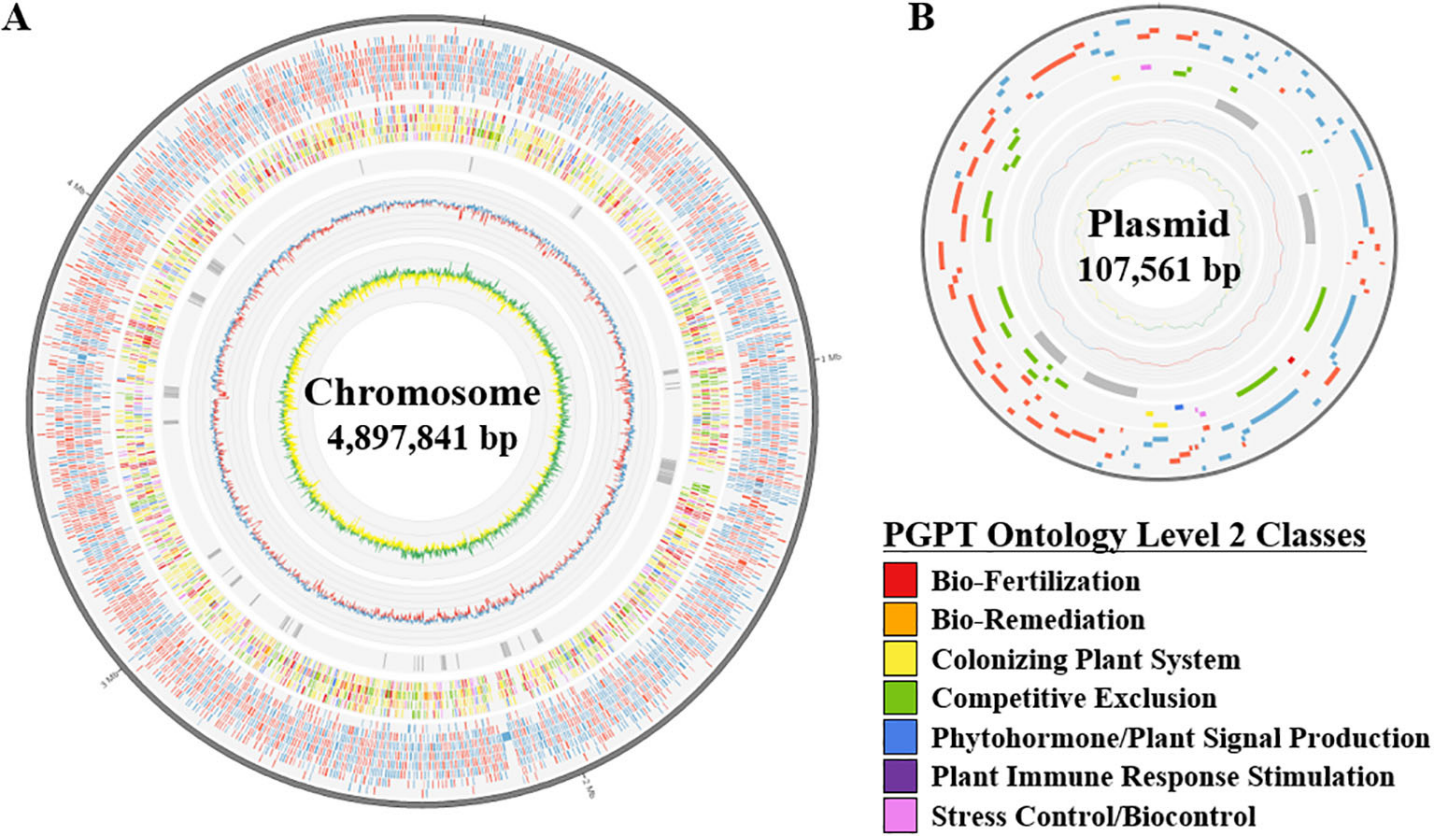
A circular diagram illustrating the genome of strain JS-SCA-14. The diagrams represent the chromosome **(A)** and plasmid **(B)** of the strain JS-SCA-14 and were organized from the outermost track to the center as follows: (i) Predicted genes were represented by blue and red segments indicating the forward and reverse strands, respectively. (ii) Genes annotated by PGPT-Pred were shown by seven functional classes of PGPT ontology level 2. (iii) 55 predicted genomic islands were indicated as grey blocks. (iv) GC content is depicted, with blue segments indicating regions above the average and red segments representing regions below the average. (v) GC skew is shown, with green segments indicating positive values (>0) and yellow segments indicating negative values (<0).

A total of 62 isolates were collected from soil samples. Strains with potential PGP capability were selected using *in vitro* assays including P-solubilization, ACC deaminase activity, siderophore and IAA production. Among them, the JS-SCA-14 strain that showed activity in two or more screening systems was selected. The complete genome sequence of the strain JS-SCA-14 was assembled into a single chromosome of 4,897,841 bp with a guanine-cytosine (GC) content of 55.73%. Gene prediction identified 4,394 protein-coding genes, 85 tRNA genes, and 25 rRNA genes within the genome sequence (**Table 1** and **Figure 1**). The predicted proteins, totaling 4,398, were classified into 22 COGs. Of these proteins, 29.32% were designated as having an unknown function or only a general functional prediction. The remaining proteins were distributed across various functional categories, with the highest counts found in genes involved in carbohydrate transport and metabolism (404), amino acid transport and metabolism (361), transcription (321), inorganic ion transport and metabolism (292), and the biogenesis of cell wall, membrane, and envelope (267) (**Supplementary Table 2**).

**Table 1:** Comparative genomic features of strains JS-SCA-14 and *Lelliottia jeotgali* PFL01^T^.

Genomic Feature^*^	1^**^	2^**^
Size of the genome assembly (bp)	4,897,841	4,603,334
GC content (%)	55.73	54.24
Protein-coding genes	4,394	4,249
CDS regions (bp)	4,324,761	4,097,455
Avg CDS (aa)	327	321
tRNA/rRNA genes	85/25	83/25
Complete BUSCOs (%)	98.86 (435/440)	99.55 (438/440)

^*^Chromosomal features only. ^**^1: JS-SCA-14, 2: *Lelliottia jeotgali* PFL01^T^.

**Figure 2:**
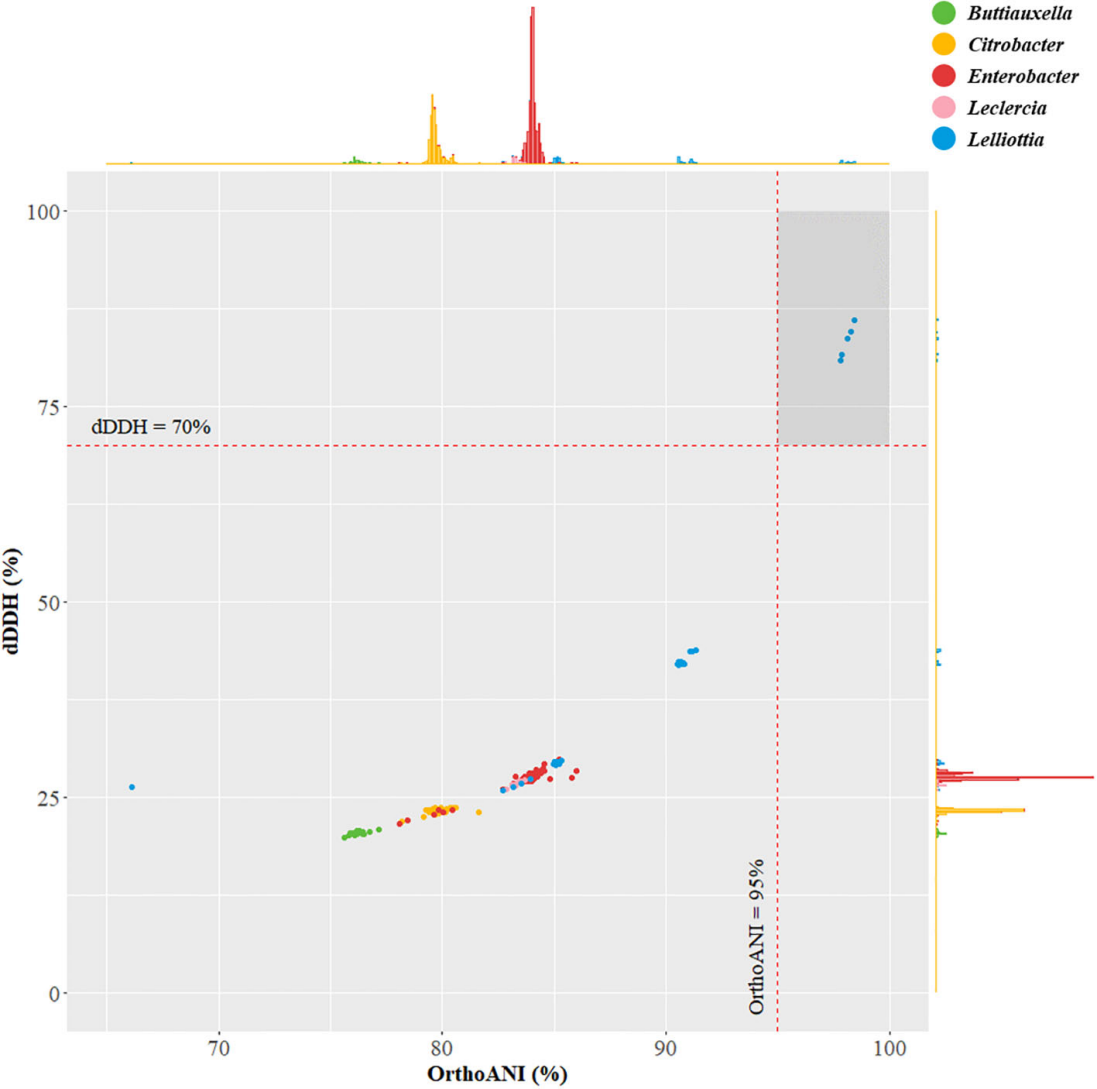
Distribution of OrthoANI and digital DNA-DNA hybridization values for strain JS-SCA-14 and closely related genera. The scatter plot presents the pairwise calculations of OrthoANI on the X-axis and dDDH (digital DNA-DNA hybridization) on the Y-axis. This plot includes strain JS-SCA-14 and a set of 957 strains from closely related genera, namely *Buttiauxella*, *Citrobacter*, *Enterobacter*, *Leclercia*, and *Lelliottia*.

For the identification of strain JS-SCA-14, an in-depth analysis of its 16S rRNA gene sequences was performed. The consensus sequence derived from the six 16S rRNA genes predicted from the genome exhibited sequence identities of 99.70%, 99.38%, and 99.38% when compared to *Lelliottia jeotgali* PFL01^T^, *Buttiauxella izardii* CCUG 35510^T^, and *L. nimipressuralis* LMG 10245^T^, respectively. According to the 16S rRNA gene analysis, 27 out of 39 hits were found in the five genera, *Buttiauxella*, *Citrobacter*, *Enterobacter*, *Leclercia*, and *Lelliottia* (**Supplementary Table 3**). A total of 957 genome sequences belonging to these five genera were selected for further genome analysis of species identification. Consequently, genome-based analyses, including dDDH and OrthoANI calculations, were conducted on a set of 957 genomes from the five closely related genera. As a result, *L. jeotgali* PFL01^T^ showed the highest OrthoANI value from the 826 out of 957 genomes that were classified with species designations (**Supplementary Table 4**). Furthermore, the genomes belonging to *Lelliottia* showed higher genomic identity than the other four closely related genera (**Supplementary Figure 1**). Meanwhile ten unclassified *Lelliottia* strains satisfied the criteria for bacterial species delineation, exhibiting 95%-96% OrthoANI and 70% dDDH (**Figure 2**) (Auch et al., 2010; Lee et al., 2016). These ten *Lelliottia* spp. were tentatively identified and proposed as novel *Lelliottia* strains (Jeong et al., 2023). Notably, they were isolated from lettuce and the surrounding soil samples, indicating a potential plant-associated habitat. Based on this comprehensive analysis, strain JS-SCA-14 was identified as novel *Lelliottia* sp.

### 3.2 Genome analysis for plant growth-promoting traits in strain JS-SCA-14

To estimate the plant growth-promoting performance *in silico*, the protein sequences of strain JS-SCA-14 were analyzed by using PGPT-Pred (Patz et al., 2021). A total of 3,009 hits were found for 2,057 PGPT accessions, where 2,168 hits for 1,688 accessions satisfied stringent cutoff of 1e-100. The total hits were grouped into seven functional categories, where 12% of hits were belonging to “bio-fertilization” class. This functional class covered key PGPTs including phosphate solubilization (173 hits), potassium solubilization (148), nitrogen acquisition (100), and iron acquisition (85) (**Supplementary Figure 2**). The results showed that strain JS-SCA-14 had ample genomic repertoire for plant growth promotion. Meanwhile four BGCs were identified from the genome encompassing 109 genes. Notably 72.48% of genes (79 out of 109) were annotated by 72 accessions of PGPT-Pred, further highlighting plant growth-promoting potential of this strain (**Supplementary Table 5**). These genes may contribute to plant growth promotion in addition to other features in the genome.

**Figure 3:**
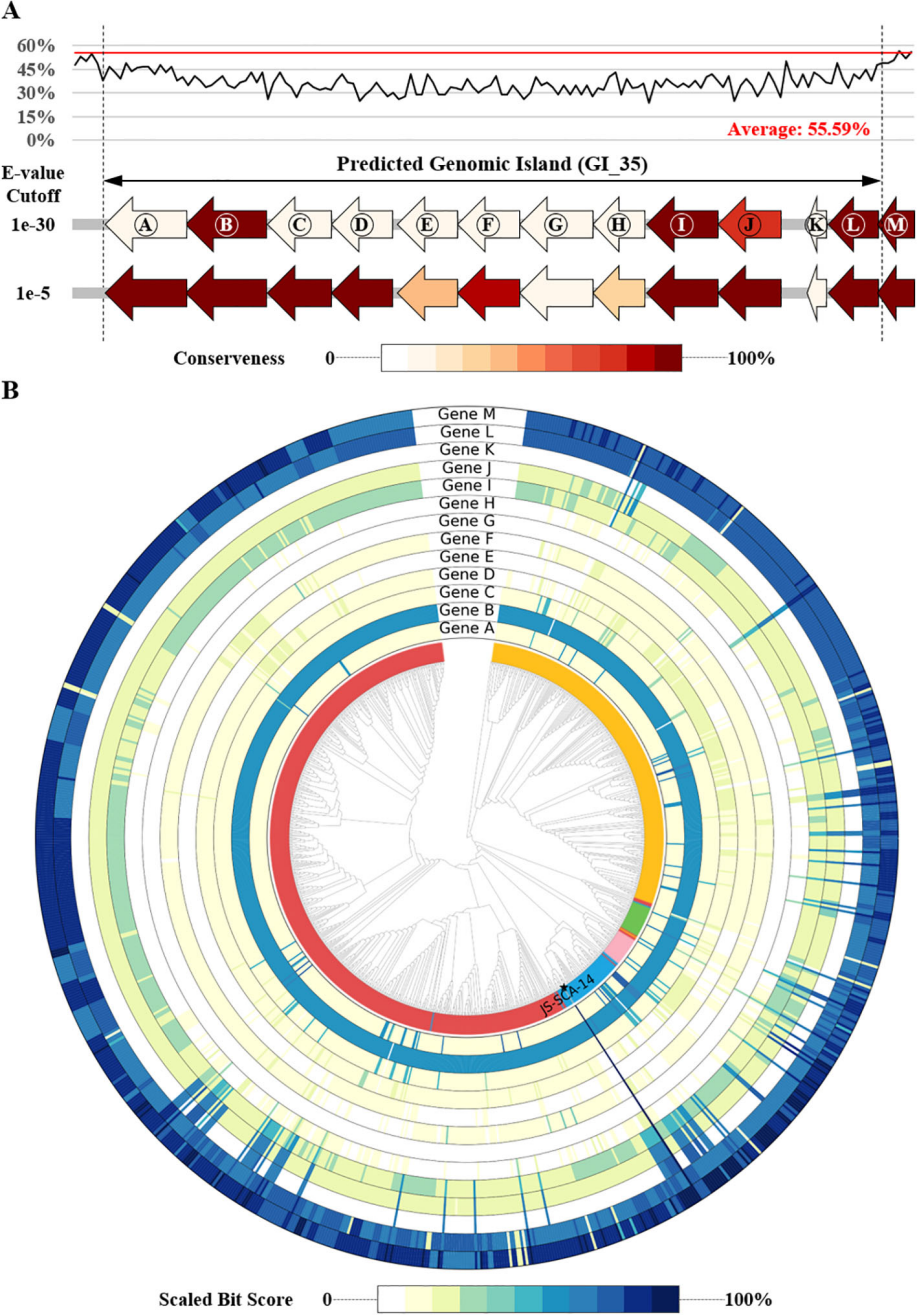
Homology distribution of genes in a genomic island of strain JS-SCA-14 across closely related genera. **(A)** Distribution of GC content (%) and predicted genes in the 13,697 bp genomic island and its flanking regions. The conserveness for each gene is represented by the ratio X/Y, where X is the number of genomes containing a hit for a given E-value cutoff and Y is the total number of genome sequences analyzed. **(B)** Relative ratio to the maximum bit score for each sequence, depicted in a color gradient at the bottom. Strain JS-SCA-14 is marked with a filled star at the terminal node.

To further search for genomic elements contributing PGP effect, genomic islands were identified from the genome sequence of strain JS-SCA-14. As a result, 55 genomic islands were predicted by IslandViewer, where more than 70% of the results (39/55) were also overlapped with those from Alien_Hunter (**Supplementary Tables 6**, **7**). One of the regions predicted by both tools (IV4_38 or GI_35) exhibited a lower GC content compared to flanking regions as well as the overall genome (**Figure 3A** and **Supplementary Tables 6**, **7**), suggesting the likelihood of horizontal transfer from an external source. None of the 957 closely related genomes showed significant orthologs for all 13 genes located on this genomic island (**Figure 3B**), further supporting the possibility of horizontal gene transfer. Using a stringent E-value threshold, only four genes (B, I, L, and M) were conserved in 97.70% or more of the analyzed genomes, while hits for eight genes (A, C, D, E, F, G, H, and K) were found in less than 10% of the genomes (**Figure 3**). Notably, this region encompassed genes involved in lipopolysaccharide (LPS) biosynthesis, including GlmM (A), RfbM (B), Wzx (I), FdtB (J), FdtA (K), RmlA (L), and RmlB (M) (Vorhölter et al., 2001). Additionally, RfbM (B) has been reported to contribute to cell wall integrity, exopolysaccharide (EPS) biosynthesis, and stress detoxification (Stroeher et al., 1997). LPS or EPS is known to confer benefits in environmental stress amelioration (Costa-Gutierrez et al., 2020), suggesting the acquisition of a beneficial trait for plant-associated bacteria. Meanwhile, a total of 3,927 BGCs were predicted from the genome sequences of strain JS-SCA-14 and the 957 closely related strains (**Supplementary Table 1**). Given their Gram-negative bacteria, the majority of the genomes (95.72%) were predicted to possess an O-antigen gene cluster. Intriguingly, they were grouped into a single component in the sequence similarity network of ribosomally synthesized and post-translationally modified peptides gene clusters (**Supplementary Figure 3**). This may suggest that the O-antigen clusters in the 958 genomes share a certain level of identity, leading to their grouping into a single component. In summary, the horizontally transferred LPS gene cluster could potentially contribute to plant growth promotion and protection against pathogenic microbes.

### 3.3 Phenotypic and chemotaxonomic characteristics of strain JS-SCA-14

The FE-SEM analysis revealed that the cells of strain JS-SCA-14 exhibited a morphology characterized by short, straight, rod-shaped structures. This morphology was similar to that of *L. jeotgali* strain PFL01^T^ (**Supplementary Figure 4**). An evaluation of 49 carbon sources indicated that JS-SCA-14 was unable to utilize glycerol, D-sorbitol, and potassium 5-ketogluconate (**Supplementary Table 8**). However, it demonstrated the ability to utilize D-sucrose, D-raffinose, and gentiobiose, unlike *L. jeotgali* strain PFL01^T^. *L. amnigenus* has been reported to be classifiable into two biological groups (Casin et al., 1986). Strains in biogroup 1 ferment D-sucrose and D-raffinose but do not ferment D-sorbitol, unlike strains in biogroup 2. According to biochemical experiments, strain JS-SCA-14 belongs to biogroup 1, while *L. jeotgali* strain PFL01^T^ belongs to biogroup 2. According to the API ZYM test results, strain JS-SCA-14 exhibited positive reactions for alkaline phosphatase, leucine arylamidase, cysteine arylamidase, acid phosphatase, naphthol-AS-BI-phosphohydrolase, α-galactosidase, β-galactosidase, and α-glucosidase (**Supplementary Table 9**). Conversely, it showed negative activity for esterase (C4), esterase lipase (C8), lipase (C14), trypsin, α-chymotrypsin, β-glucuronidase, β-glucosidase, N-acetyl-β-glucosaminidase, α-mannosidase, and α-fucosidase. In contrast to *L. jeotgali* strain PFL01^T^, which displayed negative activity for valine arylamidase, α-galactosidase, and α-glucosidase, strain JS-SCA-14 exhibited positive reactions for these enzymes. The cellular fatty acid composition of strain JS-SCA-14 included dominant fatty acids (>10%), such as C16:0 (26.18%), C16 : 1ω7c/C16 : 1ω6c (22.49%), and C18 : 1ω7c/C18 : 1ω6c (18.83%), as well as minor fatty acids (>1%) like 17:0 cyclo (9.76%), 14:0 (7.37%), 12:0 (2.98%), 19:0 cyclo ω8c (1.38), and 12:0 aldehyde (unknown, 9.97%). The cellular fatty acid profiles of both strains were not significantly different (**Supplementary Table 10**).

### 3.4 Isolation and PGP capability of strain JS-SCA-14

The colonies appeared on the isolation medium were preliminary selected based on colony morphology. A total of 62 isolates were collected, and the *Lelliottia* sp. strain JS-SCA-14 was examined for potential PGP capability using *in vitro* assay systems, including P-solubilization, ACC deaminase activity, and the production of siderophore and IAA. JS-SCA-14 did not produce IAA and showed no ACC deaminase activity. The strain exhibited dual traits of P-solubilization and siderophore production. The plate assay using PVK agar medium is routinely employed to screen for phosphate-solubilizing microorganisms. Bacteria grow on this medium and form a clear zone around the colony, indicative of their ability to convert insoluble TCP in the medium into soluble forms. The measured diameters of the JS-SCA-14 colony and the large clear/halo zone on PVK agar were 0.7 cm and 1.7 cm, respectively (**Supplementary Figure 5A**). The JS-SCA-14 strain exhibited a PSI of 3.43. Previous reports have identified genera such as *Pseudomonas*, *Enterobacter*, and *Bacillus*, *Serratia* and *Pantoea*, *Rhizobium*, *Arthrobacter*, and *Burkholderia*, and *Rahnella aquatilis* HX2, *Leclercia adecarboxylata*, and fungi like *Penicillium brevicompactum* and *Aspergillus niger*, and *Acremonium*, *Hymenella*, and *Neosartorya* as efficient phosphate solubilizers (Rawat et al., 2021). These soil microorganisms are noteworthy in integrated soil nutrient management because they improve plant nutrient acquisition in the soil (Rajwar et al., 2018; Rawat et al., 2021). Strain JS-SCA-14 demonstrated the ability to produce a halo on CAS agar medium, indicating its capacity to produce siderophore (**Supplementary Figure 5B**). Siderophores, which are secreted by PGPR, can enhance the availability of iron in the soil surrounding plant roots, thereby promoting plant growth and development. Previous studies have reported that microbial siderophores can increase both the chlorophyll content and the overall biomass of cucumber plants (Reddy, 2014). In this study, we further investigated the impact of strain JS-SCA-14 on the growth and development of tomato plants and seeds.

### 3.5 Effect of strain JS-SCA-14 on seedling germination

*Enterobacter* spp. are recognized for their wide array of PGP traits, which include nitrogen fixation, soil P-solubilization, antibiotic production, siderophore secretion, chitinase, ACC deaminase, various hydrolytic enzymes, and enhancement of soil porosity (Jha et al., 2011). Specific strains such as *E. aerogenes* sp. (NII-0907 and NII-0929), *E*. *cloacae* subsp. *cloacae* sp. (NII-0931), and *E. asburiae* sp. (NII-0934) have been reported to produce IAA, solubilize phosphorus, and produce hydrogen cyanide. These PGP capabilities make *Enterobacter* a promising candidate for promoting plant growth and development. In our study, we found that applying a fresh suspension of strain JS-SCA-14 to tomato seeds improved germination, seedling length, and vigor compared to untreated controls (**Table 2**). The root length and shoot length of tomato seeds treated with JS-SCA-14 increased by 1.28-fold and 1.24-fold, respectively, compared to the untreated control. These increases were statistically significant (*p* < 0.05 and *p* < 0.01, respectively). The germination rate of tomato seeds treated with the strain was 100%, while that of untreated tomato seeds was 94.67%. The vigor index of tomato seedlings was 551.73 ± 234.57 for the strain-treated seeds, compared to 436.13 ± 254.20 for the untreated seeds. This difference in vigor index between the treated and untreated groups was significant (*p* < 0.01). Mahdi et al. (2020) reported that quinoa seeds coated with a suspension of *Enterobacter asburiae* showed a significant increase in germination rate, ranging from 153% to 305%, compared to the control seeds. The bacterial treatment of quinoa seeds showed a significant positive effect on the total length, as well as the fresh and dry weights. The results of this study suggest that using the phosphate-solubilizing strain JS-SCA-14 as a seed inoculant not only promotes seed germination and seedling growth but also boosts productivity.

**Table 2:** Impact of strain JS-SCA-14 on tomato seed germination.

Treatment	Root length (cm)	Shoot length (cm)	Germination (%)	Vigor index
Control	2.77 ± 1.66	1.59 ± 0.96	94.67	436.13 ± 254.20
JS-SCA-14	3.54 ± 1.56^**^	1.97 ± 0.91^*^	100	551.73 ± 234.57^**^

Values are the means ± standard error from experiments with 3 replications of 25 seeds each.

Asterisks indicate significant differences with respect to the control according to the t-test (^*^ *p* < 0.05, ^**^ *p* < 0.01).

### 3.6 PGP effect of strain JS-SCA-14 on tomato plant growth

**Figure 4:**
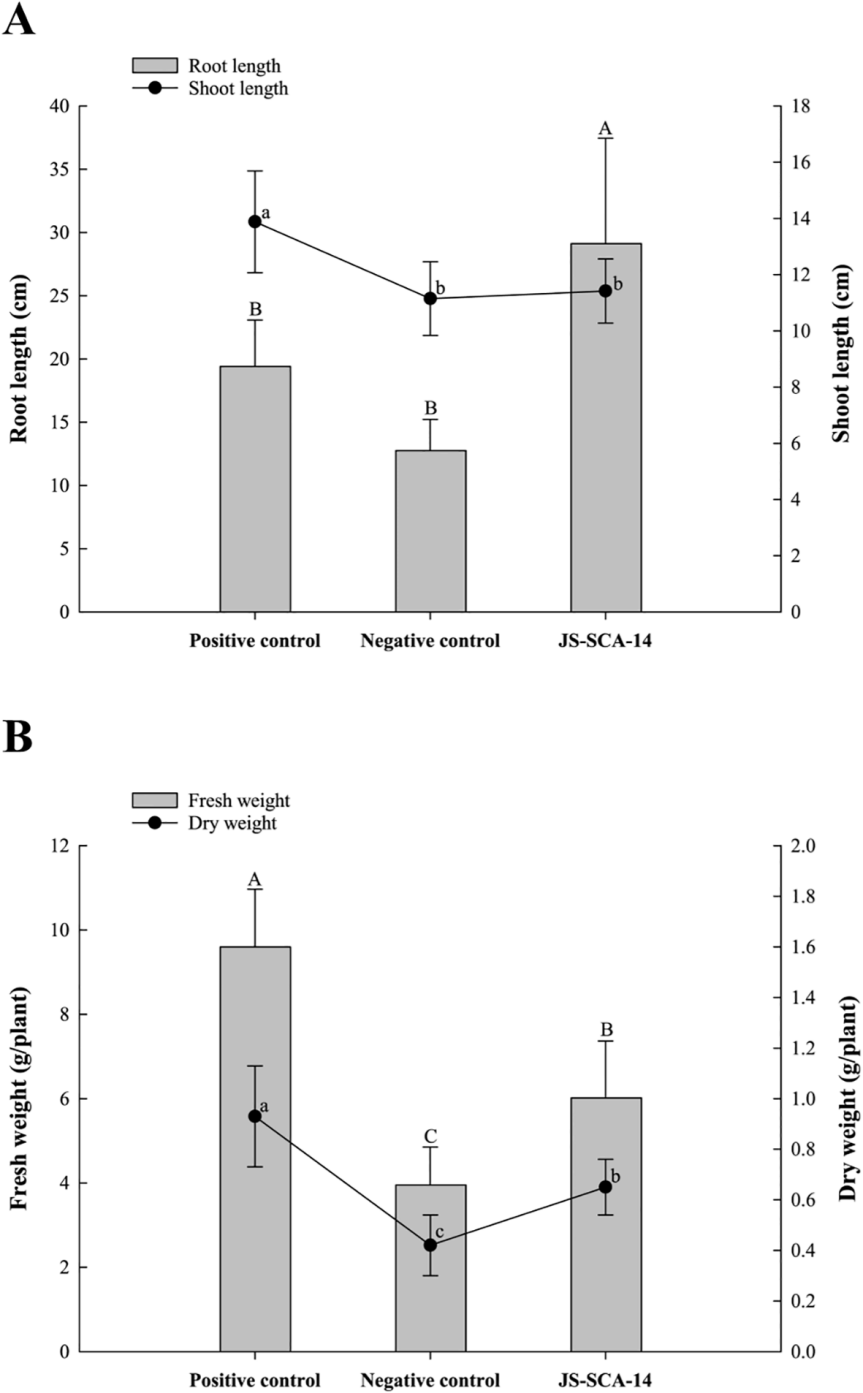
Growth promotion effects of the phosphate-solubilizing strain JS-SCA-14 on tomato plants. **(A)** Root and shoot length of tomato plants treated with the positive control, negative control, and JS-SCA-14. **(B)** Fresh and dry weight of tomato plants treated with the positive control, negative control, and JS-SCA-14. The treatments include the positive control (hoagland), the negative control (hoagland containing TCP as a P-deficient solution), and JS-SCA-14 (JS-SCA-14 + hoagland containing TCP as a P-deficient solution). The error bars indicate the standard deviation (*n* = 6). Different letters indicate significant differences between treatments using Tukey’s honest significant difference (Tukey HSD) test (*n* = 6 replicates; *p* < 0.05).

To assess the potential plant growth-promoting effects of strain JS-SCA-14 on tomato plants, various growth parameters, including shoot and root height, as well as fresh and dry weight, were measured after 30 days. Visible changes were observed in tomato plants with phosphorus deficiency. Tomato plants treated with an NH_4_H_2_PO_4_-deficient Hoagland solution and water all died. Tomato plants inoculated with Hoagland solution containing TCP as a P-deficient solution (negative control) exhibited distinct symptoms of phosphorus deficiency, such as purple discoloration of the leaves and interveinal chlorosis. Tomato plants inoculated with JS-SCA-14 along with Hoagland solution containing TCP as a P-deficient solution exhibited mild phosphorus deficiency symptoms, while tomato plants inoculated with Hoagland solution (positive control) showed no symptoms. Although mild phosphorus deficiency symptoms were observed in tomato plants treated with JS-SCA-14 together with Hoagland solution containing TCP, the inoculation of the strain JS-SCA-14 resulted in improved tomato plant growth in a greenhouse environment with phosphorus-deficient perlite. Tomato plants inoculated with TCP+JS-SCA-14 exhibited increased root length compared to both the negative control and positive control plants. The mean recorded root length was 29.12 cm for TCP+JS-SCA-14-inoculated plants, 19.42 cm for positive control plants, and 12.75 cm for negative control plants (**Figure 4A**). However, positive control plants treated with Hoagland solution showed the greatest increase in shoot length, with mean shoot lengths of 13.88 cm, 11.15 cm, and 11.42 cm for positive control, TCP+JS-SCA-14 inoculated, and negative control plants, respectively (**Figure 4A**). Despite not producing IAA, which directly influences root growth, it is thought that the JS-SCA-14 strain significantly improved the root length of tomato plants by decomposing insoluble phosphate. However, to understand the exact mechanism, additional studies are needed, including studies of genes related to phosphate-solubilizing abilities in the JS-SCA-14 and the concentration of free phosphate produced by the JS-SCA-14. The exact mechanism by which P-solubilizing agents stimulate plant growth is not fully understood but hypotheses involving the production of plant hormones and the promotion of mineral nutrient uptake are commonly proposed (Vessey, 2003; Walia et al., 2017). PSBs like *Bacillus*, *Pseudomonas*, and *Enterobacter* can convert insoluble phosphate into soluble forms through processes such as organic acid secretion, ion exchange, and chelation (Wang et al., 2022). This enhances phosphorus absorption by plant roots and improves absorption efficiency (Wu et al., 2017; Sahandi et al., 2019). Significant differences (*p* < 0.05) in fresh weight and dry weight were observed among treatments (**Figure 4B**). Positive control plants recorded the highest fresh weight (9.60 g) and dry weight (0.93 g). JS-SCA-14-inoculated tomato plants exhibited a 34.4% increase in fresh weight and a 35.4% increase in dry weight compared to negative control plants. The enhanced nutrient availability induced by phosphorus likely contributed to the increased biomass. In a greenhouse experiment, tomato plants treated with insoluble TCP and *Pantoea* sp. showed significantly improved shoot and root dry weights (Sharon et al., 2016). Soil inoculated with *E. cloacae* NG-33 strain had a pronounced impact on leaf photosynthetic rate and significantly increased maize plant height, shoot, and root biomass in both loamy and sandy soils with supplemental TCP application (Chen et al., 2022). The confirmation of phosphate-solubilizing JS-SCA-14 ability to enhance nutrient availability underscores its fundamental role in ensuring plant nutrition and promoting overall plant growth and development performance.

## 4 Conclusion

Through genome-based analyses, including dDDH and OrthoANI calculations, strain JS-SCA-14 was proposed as a novel *Lelliottia* sp. According to the API ZYM test results, strain JS-SCA-14 exhibited different patterns from a closely related strain, *L. jeotgali* PFL01^T^, in the utilization of sucrose, D-raffinose, gentiobiose, potassium 5-ketogluconate, valine arylamidase, α-galactosidase, and α-glucosidase. Strain JS-SCA-14 demonstrated an improvement in seed germination, siderophore production, and phosphate solubilization ability. Notably, tomato plants inoculated with strain JS-SCA-14 exhibited a 34.4% increase in fresh weight and a 35.4% increase in dry weight compared to the negative control plants in a plant growth promotion experiment induced by phosphate solubilization. These results demonstrate that strain JS-SCA-14 promotes plant growth and development through its phosphate solubilization ability, highlighting its potential as a bioinoculant for sustainable agriculture and plant nutrient management strategies.

## Data Availability

The datasets presented in this study can be found in online repositories. The names of the repository/repositories and accession number(s) can be found in the article/**Supplementary Material**. The complete genome sequence of *Lelliottia* sp. JS-SCA-14 was deposited in NCBI GenBank under accession numbers CP141606-CP141607 (BioProject: PRJNA1055586).
