# Supplementary material for: Plant growth-promoting effects of a novel Lelliottia sp. JS-SCA-14 and comparative genome analysis
Source: Front Plant Sci. 2024 Nov 26;15:1484616. doi: 10.3389/fpls.2024.1484616 (PMC11628249; doi:10.3389/fpls.2024.1484616)
Supplement: Supplementary file 1 [file DataSheet1.docx]

**Supplementary Data**

**Plant growth-promoting effects of a novel *Lelliottia* sp. JS-SCA-14 and comparative genome analysis**

Byeong Jun Jeon^1^, Jin-Soo Park^2^, Sung-Chul Hong^3^, Eun Ha Lee^1^, Jaeyoung Choi^4*^ and Jeong Do Kim^1*^

^1^Smart Farm Research Center, Korea Institute of Science and Technology, Gangneung 25451, Republic of Korea

^2^Natural Product Informatics Research Center, Korea Institute of Science and Technology, Gangneung 25451, Republic of Korea

^3^Department of Food Science and Biotechnology, Kunsan National University, Gunsan 54150, Republic of Korea

^4^Department of Oriental Medicine Biotechnology, College of Life Sciences, Kyung Hee University, Yongin 17104, Republic of Korea

* Correspondence: Jaeyoung Choi (jaeyoung.choi@khu.ac.kr) and Jeong Do Kim (kimjeongdo@kist.re.kr)

**Supplementary Figures**


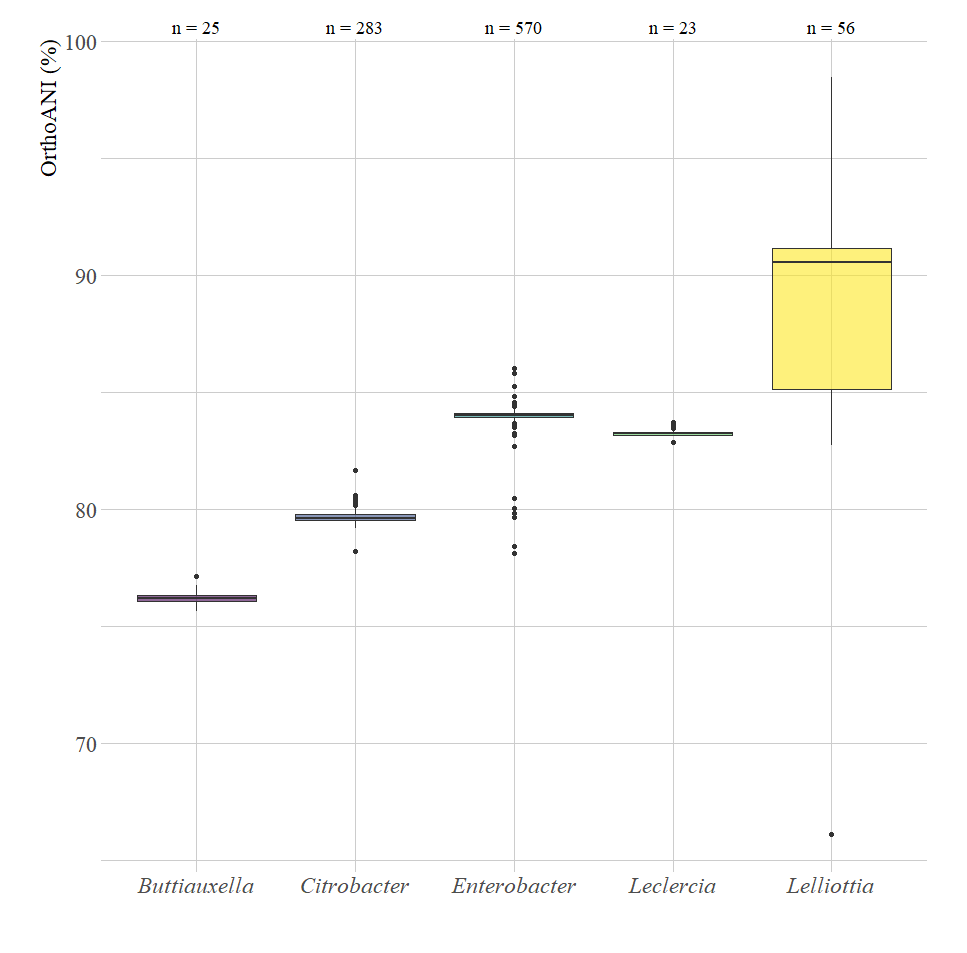
**Supplementary Figure 1. A box plot showing distribution of OrthoANI values of strain JS-SCA-14 against 957 genomes sequences belonging to the five closely related genera.**


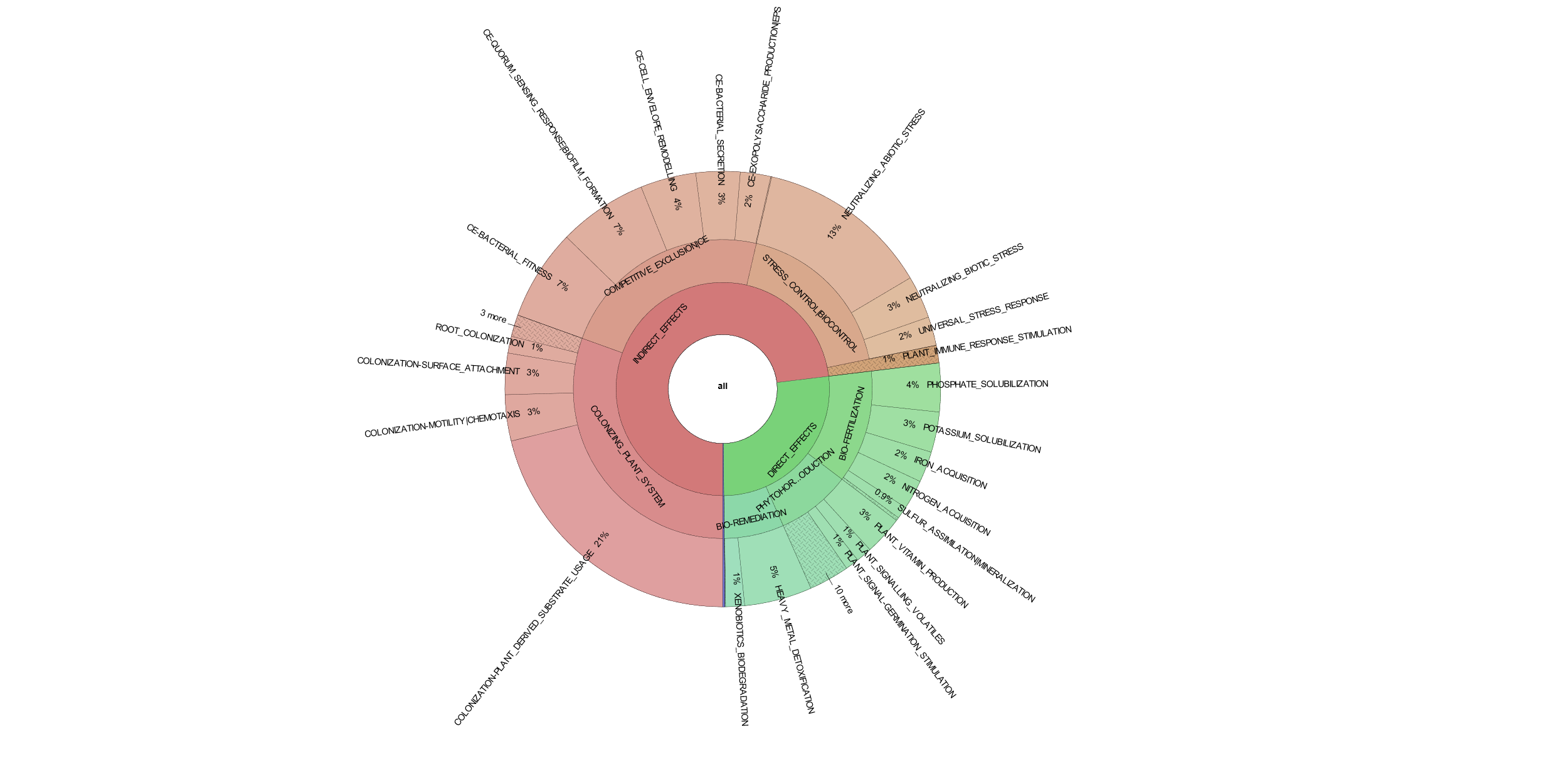


**Supplementary Figure 2. Plant growth-promoting traits (PGPTs) annotated by PGPT-Pred for strain JS-SCA-14**. Hits from PGPT-Pred with blastp+hmmer mode were grouped into seven functional classes on PGPT ontology level 2 and 41 classes on level 3.

**
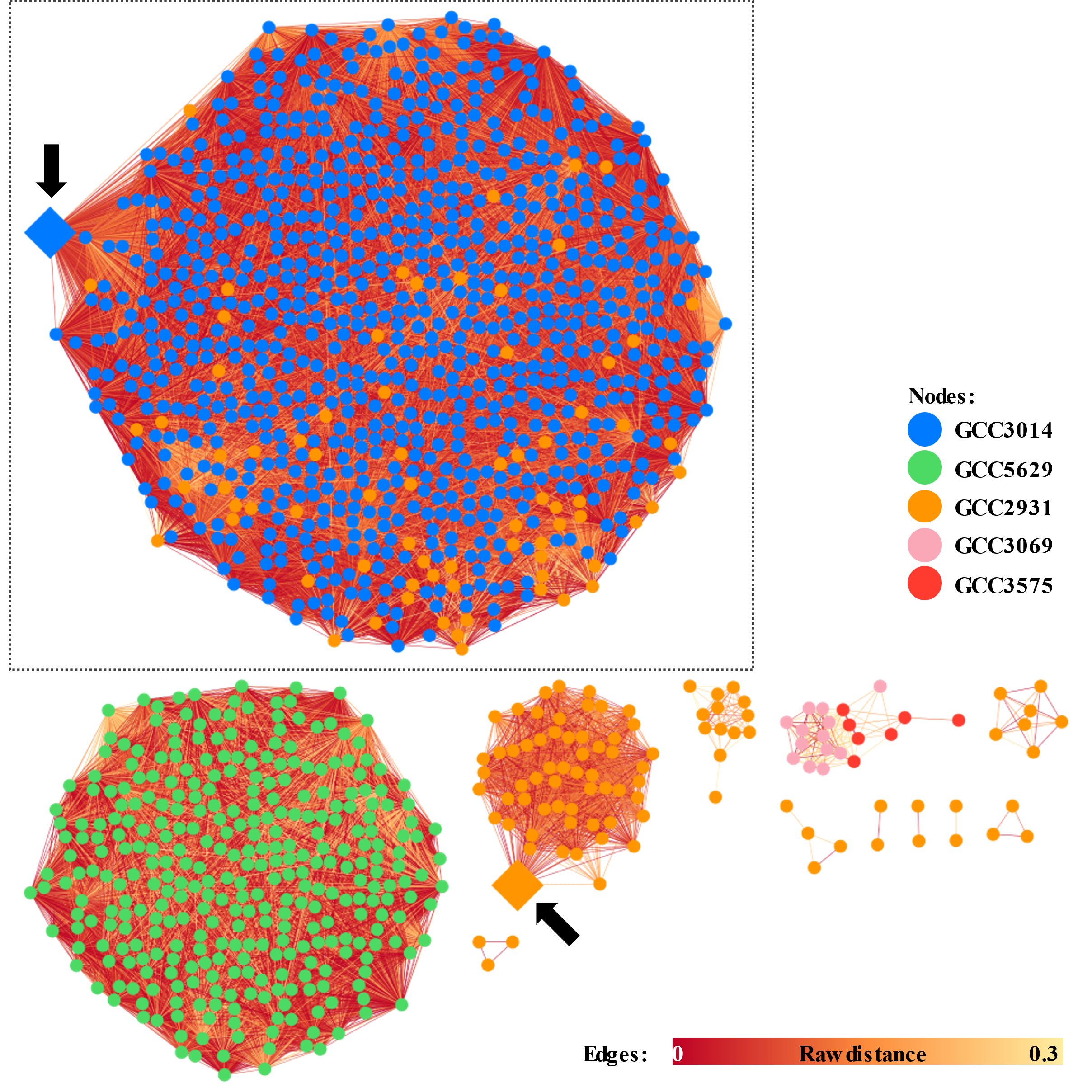
**

**Supplementary Figure 3. Sequence similarity network of RiPP BGCs generated by BiG-SCAPE.** A total of 1,372 RiPP BGCs, with 18 singletons excluded, were identified from strain JS-SCA-14 along with 957 closely related genomes and were visually represented in a similarity network. Nodes were color-coded based on the gene family clans (GCCs) they belong to, using five distinct colors. Edges were shown in a color gradient representing the raw distance obtained from BiG-SCAPE analysis. The network component encompassing thiopeptide BGCs was demarcated with a dashed box. Additionally, two BGCs identified from JS-SCA-14 were highlighted as diamonds and indicated by arrows.


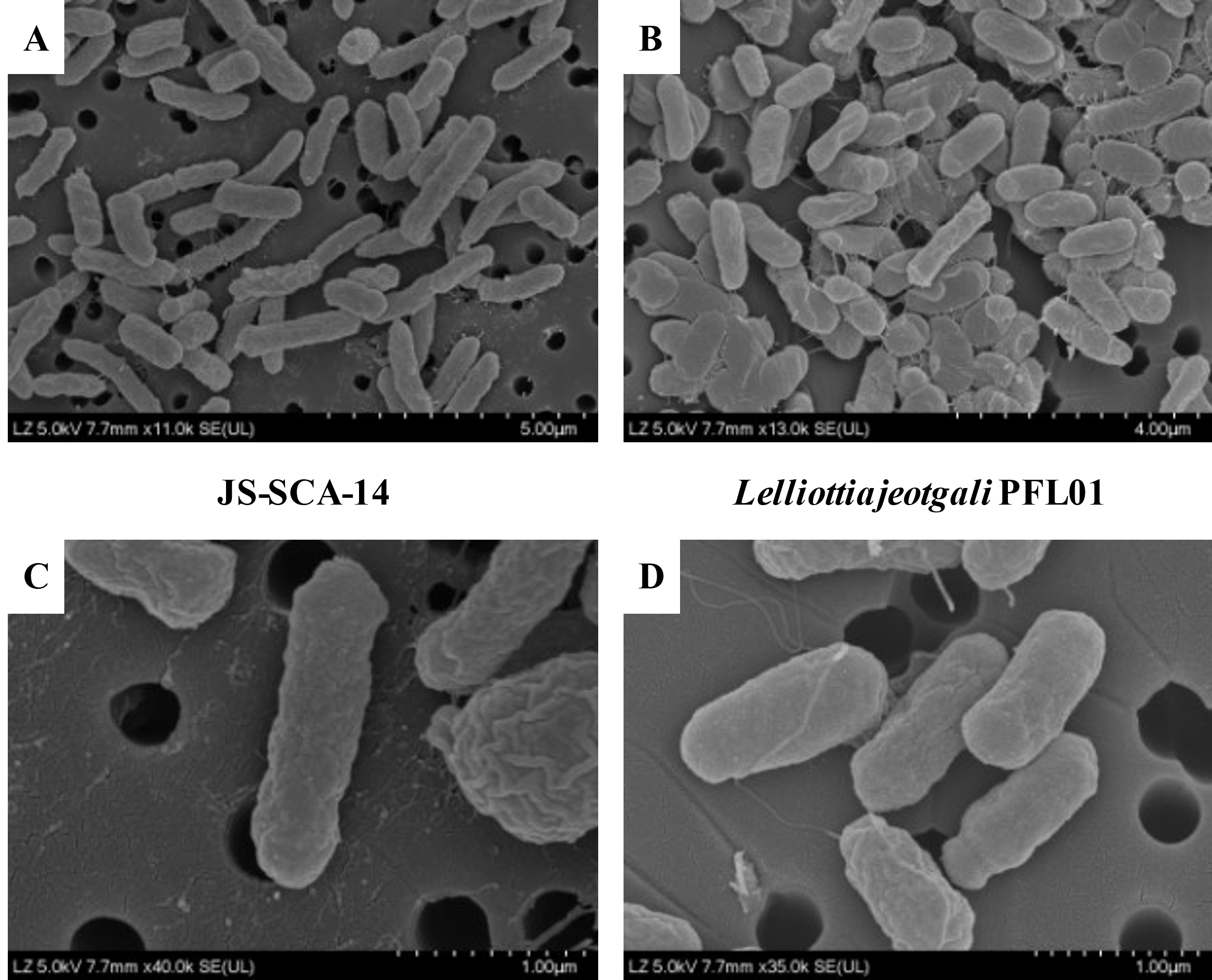


**Supplementary Figure 4. Scanning electron microscopy image of strain JS-SCA-14 and PFL01 grown in tryptic soy broth at 28°C for 2 days. (A and B)** Cells of both strains were observed to be short and straight rod-shaped using FE-SEM at 5 kV. **(C and D)** Images of both strains at a higher magnification.


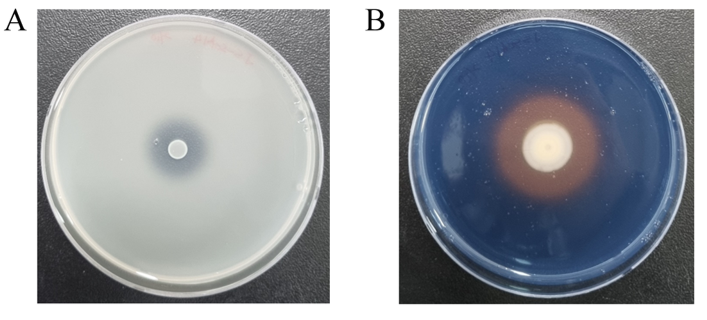


**Supplementary Figure 5. Plant growth-promoting activities of strain JS-SCA-14**.
**(A)** Pikovskaya’s agar plate demonstrating phosphate solubilization by strain JS-SCA-14.
**(B)** Chromo-azurol S plate showing siderophore production by strain JS-SCA-14.

**Supplementary Tables**

**Supplementary Table 1**. List of 957 closely related species belonging to the genera *Buttiauxella*, *Citrobacter*, *Enterobacter*, *Leclercia*, and *Lelliottia*

| **Species** | **Accession** | **Species** | **Accession** |
| --- | --- | --- | --- |
| *Buttiauxella agresti* | GCF_000735355.1 | *Enterobacter hormaechei* | GCF_009738085.1 |
|  | GCF_013234275.1 |  | GCF_009905155.1 |
|  | GCF_900446255.1 |  | GCF_009930835.1 |
| *Buttiauxella brennerae* | GCF_001654925.1 |  | GCF_009930935.1 |
| *Buttiauxella ferragutiae* | GCF_001654915.1 |  | GCF_011044215.1 |
|  | GCF_021282485.1 |  | GCF_011045335.1 |
|  | GCF_022637515.1 |  | GCF_011045355.1 |
| *Buttiauxella gaviniae* | GCF_001654835.1 |  | GCF_012562255.1 |
| *Buttiauxella izardii* | GCF_003601925.1 |  | GCF_012975085.1 |
| *Buttiauxella massiliensis* | GCF_902500225.1 |  | GCF_013046845.1 |
| *Buttiauxella noackiae* | GCF_000737905.1 |  | GCF_013624605.1 |
|  | GCF_001654865.1 |  | GCF_013624995.1 |
|  | GCF_020216825.1 |  | GCF_013625115.1 |
| *Buttiauxella warmboldiae* | GCF_003818135.1 |  | GCF_013625265.1 |
| *Buttiauxella* sp. | GCF_003675305.1 |  | GCF_013625435.1 |
|  | GCF_004361975.1 |  | GCF_013625575.1 |
|  | GCF_004366275.1 |  | GCF_013625795.1 |
|  | GCF_006376615.1 |  | GCF_013629235.1 |
|  | GCF_014189855.1 |  | GCF_013629455.1 |
|  | GCF_021282405.1 |  | GCF_013629675.1 |
|  | GCF_021282435.1 |  | GCF_013635255.1 |
|  | GCF_021283185.1 |  | GCF_013635495.1 |
|  | GCF_022134725.1 |  | GCF_013707135.1 |
|  | GCF_024807715.1 |  | GCF_013708455.1 |
|  | GCF_027594725.1 |  | GCF_013726395.1 |
| *Citrobacter amalonaticus* | GCF_000981805.1 |  | GCF_013731155.1 |
|  | GCF_001558935.2 |  | GCF_013734275.1 |
|  | GCF_001559075.2 |  | GCF_013734915.1 |
|  | GCF_006965505.1 |  | GCF_013738375.1 |
|  | GCF_015353115.1 |  | GCF_013739595.1 |
|  | GCF_018323885.1 |  | GCF_013740655.1 |
|  | GCF_019645955.1 |  | GCF_013742375.1 |
|  | GCF_020099335.1 |  | GCF_013743295.1 |
|  | GCF_025723225.1 |  | GCF_013743755.1 |
|  | GCF_900079785.1 |  | GCF_013743935.1 |
|  | GCF_900079795.1 |  | GCF_013744415.1 |
| *Citrobacter arsenatis* | GCF_004353845.1 |  | GCF_013747035.1 |
| *Citrobacter braakii* | GCF_002208845.2 |  | GCF_013873515.1 |
|  | GCF_009648935.1 |  | GCF_013873655.1 |
|  | GCF_016894145.1 |  | GCF_013873945.1 |
|  | GCF_019048045.1 |  | GCF_014109705.1 |
|  | GCF_019048805.1 |  | GCF_014117285.1 |
|  | GCF_019270195.1 |  | GCF_014333255.1 |
|  | GCF_019270215.1 |  | GCF_014333275.1 |
|  | GCF_019270235.1 |  | GCF_014771415.1 |
|  | GCF_019270255.1 |  | GCF_015137995.1 |
|  | GCF_019270275.1 |  | GCF_015238615.1 |
|  | GCF_019270295.1 |  | GCF_015535055.1 |
|  | GCF_019270315.1 |  | GCF_015910205.1 |
|  | GCF_019270335.1 |  | GCF_015910225.1 |
|  | GCF_019270355.1 |  | GCF_015910245.1 |
|  | GCF_019270375.1 |  | GCF_015910265.1 |
|  | GCF_019270455.1 |  | GCF_015910285.1 |
|  | GCF_019270575.1 |  | GCF_015910305.1 |
|  | GCF_020034515.1 |  | GCF_015910325.1 |
|  | GCF_026626145.1 |  | GCF_015910345.1 |
|  | GCF_029001825.1 |  | GCF_015910365.1 |
|  | GCF_029634665.1 |  | GCF_015910385.1 |
|  | GCF_029715665.1 |  | GCF_015910425.1 |
|  | GCF_029716315.1 |  | GCF_016126855.1 |
|  | GCF_029716485.1 |  | GCF_016725825.1 |
|  | GCF_029716845.1 |  | GCF_016745355.1 |
|  | GCF_029717445.1 |  | GCF_016767735.1 |
|  | GCF_029717465.1 |  | GCF_016767755.1 |
|  | GCF_029717665.1 |  | GCF_016864035.1 |
|  | GCF_029717885.1 |  | GCF_016864135.1 |
|  | GCF_029717925.1 |  | GCF_016864155.1 |
|  | GCF_029717945.1 |  | GCF_016939555.1 |
|  | GCF_029718275.1 |  | GCF_017161605.1 |
|  | GCF_029718295.1 |  | GCF_017161725.1 |
|  | GCF_029718425.1 |  | GCF_017161745.1 |
|  | GCF_029718465.1 |  | GCF_017161765.1 |
|  | GCF_029718585.1 |  | GCF_017161785.1 |
|  | GCF_029719125.1 |  | GCF_017161805.1 |
|  | GCF_029719805.1 |  | GCF_017161825.1 |
|  | GCF_030166475.1 |  | GCF_017161845.1 |
| *Citrobacter cronae* | GCF_016893685.1 |  | GCF_017161865.1 |
|  | GCF_016893705.1 |  | GCF_017310485.1 |
|  | GCF_016893745.1 |  | GCF_018255815.1 |
|  | GCF_016893845.1 |  | GCF_019047945.1 |
|  | GCF_016893885.1 |  | GCF_019048245.1 |
|  | GCF_016893985.1 |  | GCF_019048625.1 |
|  | GCF_016894025.1 |  | GCF_019356035.1 |
|  | GCF_016894045.1 |  | GCF_019458525.1 |
|  | GCF_016894065.1 |  | GCF_019537235.1 |
|  | GCF_016894085.1 |  | GCF_019537255.1 |
|  | GCF_016894105.1 |  | GCF_019537275.1 |
|  | GCF_022558585.1 |  | GCF_019537295.1 |
| *Citrobacter farmeri* | GCF_019048065.1 |  | GCF_019537315.1 |
|  | GCF_019803045.1 |  | GCF_019537335.1 |
| *Citrobacter freundii* | GCF_000648515.1 |  | GCF_020695365.1 |
|  | GCF_000783995.2 |  | GCF_020695485.1 |
|  | GCF_001022155.1 |  | GCF_020695585.1 |
|  | GCF_001022275.1 |  | GCF_020695605.1 |
|  | GCF_001718055.1 |  | GCF_020695685.1 |
|  | GCF_001922445.1 |  | GCF_020695705.1 |
|  | GCF_002215385.1 |  | GCF_020695725.1 |
|  | GCF_002786865.1 |  | GCF_020861325.1 |
|  | GCF_002796505.1 |  | GCF_021397695.1 |
|  | GCF_003019835.1 |  | GCF_021398115.1 |
|  | GCF_003665535.1 |  | GCF_021491615.1 |
|  | GCF_003665595.1 |  | GCF_021491635.1 |
|  | GCF_003665615.1 |  | GCF_021496135.1 |
|  | GCF_003665635.1 |  | GCF_021497625.1 |
|  | GCF_003665655.1 |  | GCF_021545705.1 |
|  | GCF_003665675.1 |  | GCF_022023895.1 |
|  | GCF_003812325.1 |  | GCF_022023915.1 |
|  | GCF_003812345.1 |  | GCF_022023955.1 |
|  | GCF_004103775.1 |  | GCF_023375605.1 |
|  | GCF_004792475.1 |  | GCF_023547225.1 |
|  | GCF_008931485.1 |  | GCF_023547485.1 |
|  | GCF_008931505.1 |  | GCF_023612855.1 |
|  | GCF_009857035.1 |  | GCF_023612875.1 |
|  | GCF_010365585.1 |  | GCF_023734795.1 |
|  | GCF_011064845.1 |  | GCF_023734815.1 |
|  | GCF_012955545.1 |  | GCF_023734835.1 |
|  | GCF_013085885.1 |  | GCF_023734855.1 |
|  | GCF_013371705.1 |  | GCF_023734875.1 |
|  | GCF_013389635.1 |  | GCF_023734895.1 |
|  | GCF_013705425.1 |  | GCF_023734915.1 |
|  | GCF_013706575.1 |  | GCF_023734935.1 |
|  | GCF_013707835.1 |  | GCF_023734955.1 |
|  | GCF_013724765.1 |  | GCF_023734975.1 |
|  | GCF_013724985.1 |  | GCF_023734995.1 |
|  | GCF_013725595.1 |  | GCF_023735015.1 |
|  | GCF_013726215.1 |  | GCF_023735035.1 |
|  | GCF_013726615.1 |  | GCF_023735055.1 |
|  | GCF_013727455.1 |  | GCF_023735075.1 |
|  | GCF_013731955.1 |  | GCF_023735155.1 |
|  | GCF_013739375.1 |  | GCF_023735195.1 |
|  | GCF_013740875.1 |  | GCF_023735215.1 |
|  | GCF_013741395.1 |  | GCF_023735235.1 |
|  | GCF_013748235.1 |  | GCF_023735255.1 |
|  | GCF_013748855.1 |  | GCF_023735275.1 |
|  | GCF_013751655.1 |  | GCF_023735295.1 |
|  | GCF_013751895.1 |  | GCF_023735315.1 |
|  | GCF_013784225.1 |  | GCF_023735335.1 |
|  | GCF_013784645.1 |  | GCF_023735355.1 |
|  | GCF_013785085.1 |  | GCF_023735375.1 |
|  | GCF_013785525.1 |  | GCF_023735395.1 |
|  | GCF_013786025.1 |  | GCF_023735435.1 |
|  | GCF_013786145.1 |  | GCF_023735475.1 |
|  | GCF_013786205.1 |  | GCF_023735495.1 |
|  | GCF_013790265.1 |  | GCF_023735515.1 |
|  | GCF_013791615.1 |  | GCF_023735535.1 |
|  | GCF_013792125.1 |  | GCF_023735615.1 |
|  | GCF_013792365.1 |  | GCF_023735695.1 |
|  | GCF_013798115.1 |  | GCF_023735715.1 |
|  | GCF_013798355.1 |  | GCF_023735735.1 |
|  | GCF_013801705.1 |  | GCF_023735755.1 |
|  | GCF_013815045.1 |  | GCF_023735775.1 |
|  | GCF_013821695.1 |  | GCF_023735795.1 |
|  | GCF_013828645.1 |  | GCF_023735815.1 |
|  | GCF_013882995.1 |  | GCF_023735835.1 |
|  | GCF_013883595.1 |  | GCF_023735855.1 |
|  | GCF_013899365.1 |  | GCF_023735875.1 |
|  | GCF_014169975.1 |  | GCF_023735895.1 |
|  | GCF_014170055.1 |  | GCF_023735915.1 |
|  | GCF_014189285.1 |  | GCF_023735935.1 |
|  | GCF_014189355.1 |  | GCF_023735955.1 |
|  | GCF_014189375.1 |  | GCF_023735975.1 |
|  | GCF_014725915.1 |  | GCF_023735995.1 |
|  | GCF_016939615.1 |  | GCF_023736015.1 |
|  | GCF_017086505.1 |  | GCF_023805415.1 |
|  | GCF_020809005.1 |  | GCF_023897035.1 |
|  | GCF_022430505.1 |  | GCF_024138915.1 |
|  | GCF_022646275.1 |  | GCF_024182005.1 |
|  | GCF_023330605.1 |  | GCF_024182025.1 |
|  | GCF_023612475.1 |  | GCF_024917315.1 |
|  | GCF_023658085.1 |  | GCF_024917395.1 |
|  | GCF_024138935.1 |  | GCF_024917635.1 |
|  | GCF_026240835.1 |  | GCF_024917775.1 |
|  | GCF_026240875.1 |  | GCF_024918215.1 |
|  | GCF_026526975.1 |  | GCF_024918255.1 |
|  | GCF_027595105.1 |  | GCF_024918795.1 |
|  | GCF_029716005.1 |  | GCF_025369895.1 |
|  | GCF_029717145.1 |  | GCF_025398395.2 |
|  | GCF_029717485.1 |  | GCF_025398715.1 |
|  | GCF_029717865.1 |  | GCF_026015945.1 |
|  | GCF_029717985.1 |  | GCF_026240655.1 |
|  | GCF_029718045.1 |  | GCF_027271235.1 |
|  | GCF_029718965.1 |  | GCF_027595125.1 |
|  | GCF_029719365.1 |  | GCF_027886505.1 |
|  | GCF_029719765.1 |  | GCF_027886525.1 |
|  | GCF_030007575.1 |  | GCF_027886545.1 |
|  | GCF_030007795.1 |  | GCF_028622735.1 |
|  | GCF_030035205.1 |  | GCF_028743675.1 |
|  | GCF_030064365.1 |  | GCF_028872235.1 |
|  | GCF_030122495.1 |  | GCF_028891505.1 |
|  | GCF_030177735.1 |  | GCF_028898905.1 |
|  | GCF_900520335.1 |  | GCF_029207635.1 |
|  | GCF_900520375.1 |  | GCF_029226365.1 |
|  | GCF_902387635.1 |  | GCF_029719585.1 |
|  | GCF_904425445.1 |  | GCF_029719845.1 |
|  | GCF_904859905.1 |  | GCF_029719865.1 |
|  | GCF_905218895.2 |  | GCF_029721335.1 |
|  | GCF_905219065.2 |  | GCF_029948145.1 |
|  | GCF_905232395.2 |  | GCF_030286165.1 |
|  | GCF_905232515.2 |  | GCF_030324195.1 |
|  | GCF_905329885.2 |  | GCF_030324215.1 |
|  | GCF_905338045.2 |  | GCF_900177445.1 |
| *Citrobacter freundii* complex sp. | GCF_002880615.1 |  | GCF_900497145.1 |
|  | GCF_002903215.1 |  | GCF_900635705.1 |
| *Citrobacter koseri* | GCF_000018045.1 |  | GCF_902164655.1 |
|  | GCF_002208985.1 |  | GCF_902164705.1 |
|  | GCF_002393245.1 |  | GCF_902166375.1 |
|  | GCF_002947035.1 |  | GCF_902166405.1 |
|  | GCF_002947675.1 |  | GCF_902166455.1 |
|  | GCF_003812405.1 |  | GCF_902166485.1 |
|  | GCF_008693945.1 |  | GCF_902166535.1 |
|  | GCF_012955585.1 |  | GCF_902166595.1 |
|  | GCF_014333315.1 |  | GCF_902166715.1 |
|  | GCF_014679775.1 |  | GCF_902166805.1 |
|  | GCF_016128275.1 | *Enterobacter hormaechei* subsp. *hoffmannii* | GCF_000750225.1 |
|  | GCF_018502305.1 |  | GCF_000750275.1 |
|  | GCF_019443705.1 |  | GCF_001729745.1 |
|  | GCF_029025745.1 |  | GCF_002968455.1 |
|  | GCF_905337805.2 |  | GCF_009834325.1 |
|  | GCF_905337845.2 |  | GCF_010319625.1 |
| *Citrobacter pasteurii* | GCF_003665575.1 |  | GCF_000814205.1 |
|  | GCF_019047765.1 |  | GCF_025266795.1 |
| *Citrobacter portucalensis* | GCF_001281005.1 | *Enterobacter hormaechei* subsp. *oharae* | GCF_001729705.1 |
|  | GCF_002215605.1 |  | GCF_020097195.1 |
|  | GCF_004801555.1 | *Enterobacter hormaechei* subsp. *steigerwaltii* | GCF_000807425.2 |
|  | GCF_008086465.1 |  | GCF_000814125.3 |
|  | GCF_008693605.1 |  | GCF_001729725.1 |
|  | GCF_009730355.1 |  | GCF_007556795.1 |
|  | GCF_010319905.2 |  | GCF_015535675.1 |
|  | GCF_015134935.1 |  | GCF_019355415.1 |
|  | GCF_015135075.1 |  | GCF_023023665.1 |
|  | GCF_015135215.1 |  | GCF_024218835.1 |
|  | GCF_015137095.1 |  | GCF_025244885.1 |
|  | GCF_015137255.1 |  | GCF_025311535.1 |
|  | GCF_015138175.1 |  | GCF_025311555.1 |
|  | GCF_016406035.3 |  | GCF_025311595.1 |
|  | GCF_018279205.1 |  | GCF_027920365.1 |
|  | GCF_019265345.1 |  | GCF_900322715.1 |
|  | GCF_019265365.1 | *Enterobacter hormaechei* subsp. *xiangfangensis* | GCF_000807405.2 |
|  | GCF_021228875.1 |  | GCF_000814225.1 |
|  | GCF_021496985.1 |  | GCF_001729785.1 |
|  | GCF_022354565.1 |  | GCF_003254805.1 |
|  | GCF_023374935.1 |  | GCF_003382725.1 |
|  | GCF_023650815.1 |  | GCF_003586025.1 |
|  | GCF_029719185.1 |  | GCF_003964795.2 |
|  | GCF_029719205.1 |  | GCF_003999755.1 |
|  | GCF_029719225.1 |  | GCF_014931695.1 |
|  | GCF_030179595.1 |  | GCF_022376815.1 |
|  | GCF_900636015.1 |  | GCF_024585325.1 |
|  | GCF_902381795.1 |  | GCF_026109755.1 |
| *Citrobacter rodentium* | GCF_000027085.1 |  | GCF_028752775.1 |
|  | GCF_021278985.1 |  | GCF_029639565.1 |
| *Citrobacter sedlakii* | GCF_018128425.1 |  | GCF_030062075.1 |
| *Citrobacter tructae* | GCF_004684345.1 | *Enterobacter huaxiensis* | GCF_003594935.2 |
| *Citrobacter werkmanii* | GCF_002025225.1 | *Enterobacter kobei* | GCF_000286275.1 |
|  | GCF_002386385.1 |  | GCF_001729765.1 |
|  | GCF_003665555.1 |  | GCF_003665375.1 |
|  | GCF_008693645.1 |  | GCF_008365235.1 |
|  | GCF_020271725.1 |  | GCF_008931545.1 |
|  | GCF_020341495.1 |  | GCF_011604705.1 |
|  | GCF_902388105.1 |  | GCF_014041955.1 |
| *Citrobacter youngae* | GCF_024662035.1 |  | GCF_015135595.1 |
|  | GCF_900638065.1 |  | GCF_015135855.1 |
| *Citrobacter* sp. | GCF_001559235.2 |  | GCF_015136055.1 |
|  | GCF_002934585.1 |  | GCF_015137465.1 |
|  | GCF_003204265.1 |  | GCF_022559745.1 |
|  | GCF_004322975.1 |  | GCF_023023125.1 |
|  | GCF_005406305.1 |  | GCF_023023705.1 |
|  | GCF_006385635.1 |  | GCF_023333675.1 |
|  | GCF_009649895.1 |  | GCF_023547315.1 |
|  | GCF_009649935.1 |  | GCF_023558885.1 |
|  | GCF_009905315.1 |  | GCF_023612495.1 |
|  | GCF_011602505.1 |  | GCF_025398875.1 |
|  | GCF_012524235.2 |  | GCF_026240695.1 |
|  | GCF_013458095.1 |  | GCF_027595935.1 |
|  | GCF_013615435.1 |  | GCF_028891545.1 |
|  | GCF_013727235.1 | *Enterobacter ludwigii* | GCF_000239975.1 |
|  | GCF_013739755.1 |  | GCF_000512375.1 |
|  | GCF_013741155.1 |  | GCF_001029645.1 |
|  | GCF_013747275.1 |  | GCF_001750725.1 |
|  | GCF_013747755.1 |  | GCF_002025685.1 |
|  | GCF_013781945.1 |  | GCF_005518115.1 |
|  | GCF_013781985.1 |  | GCF_005848825.1 |
|  | GCF_013783065.1 |  | GCF_005890075.1 |
|  | GCF_013784065.1 |  | GCF_006385915.1 |
|  | GCF_013786345.1 |  | GCF_011463635.1 |
|  | GCF_013791465.1 |  | GCF_013375975.1 |
|  | GCF_013797345.1 |  | GCF_019930965.1 |
|  | GCF_013797615.1 |  | GCF_020099215.1 |
|  | GCF_013814485.1 |  | GCF_020783215.1 |
|  | GCF_013816515.1 |  | GCF_020783235.1 |
|  | GCF_013816795.1 |  | GCF_020783255.1 |
|  | GCF_013836145.1 |  | GCF_022832735.1 |
|  | GCF_013889015.1 |  | GCF_028198205.1 |
|  | GCF_013889875.1 |  | GCF_029718255.1 |
|  | GCF_013890155.1 |  | GCF_029718315.1 |
|  | GCF_013890595.1 |  | GCF_029718885.1 |
|  | GCF_015167655.1 |  | GCF_029720995.1 |
|  | GCF_016811995.1 |  | GCF_029721015.1 |
|  | GCF_016906145.1 |  | GCF_029834565.1 |
|  | GCF_016906165.1 | *Enterobacter mori* | GCF_015708675.1 |
|  | GCF_019382495.1 |  | GCF_018638795.1 |
|  | GCF_019382615.1 |  | GCF_019203945.1 |
|  | GCF_019382635.1 |  | GCF_020494085.1 |
|  | GCF_023516235.1 |  | GCF_022014715.1 |
|  | GCF_024181025.1 |  | GCF_025244905.1 |
|  | GCF_024181045.1 | *Enterobacter oligotrophicus* | GCF_009176645.1 |
|  | GCF_027919895.1 | *Enterobacter quasiroggenkampii* | GCF_029542725.1 |
|  | GCF_029338175.1 | *Enterobacter roggenkampii* | GCF_000807415.2 |
| *Enterobacter asburiae* | GCF_000632395.1 |  | GCF_001729805.1 |
|  | GCF_001022095.1 |  | GCF_002007805.1 |
|  | GCF_001521715.1 |  | GCF_002211685.1 |
|  | GCF_003940765.1 |  | GCF_003812145.1 |
|  | GCF_007035645.1 |  | GCF_004138605.1 |
|  | GCF_007035805.1 |  | GCF_004684365.1 |
|  | GCF_009755685.1 |  | GCF_009184765.2 |
|  | GCF_011396735.1 |  | GCF_013389415.1 |
|  | GCF_013740195.1 |  | GCF_013403545.1 |
|  | GCF_013782005.1 |  | GCF_013635755.1 |
|  | GCF_013784425.1 |  | GCF_013708155.1 |
|  | GCF_015168595.1 |  | GCF_013725995.1 |
|  | GCF_016027695.1 |  | GCF_013728935.1 |
|  | GCF_016403185.1 |  | GCF_013733395.1 |
|  | GCF_017347425.1 |  | GCF_013742615.1 |
|  | GCF_018394395.1 |  | GCF_013927185.1 |
|  | GCF_019048485.1 |  | GCF_014490825.1 |
|  | GCF_019428505.1 |  | GCF_014490845.1 |
|  | GCF_019968785.1 |  | GCF_014524505.1 |
|  | GCF_019968865.1 |  | GCF_015136315.1 |
|  | GCF_019973695.1 |  | GCF_016756655.1 |
|  | GCF_022570715.1 |  | GCF_016864055.1 |
|  | GCF_023023045.1 |  | GCF_018596855.2 |
|  | GCF_023023145.1 |  | GCF_019047025.1 |
|  | GCF_023023265.1 |  | GCF_019394605.1 |
|  | GCF_023101705.1 |  | GCF_020881955.1 |
|  | GCF_024599655.1 |  | GCF_021347605.1 |
|  | GCF_024917675.1 |  | GCF_023023065.1 |
|  | GCF_025999975.1 |  | GCF_023023685.1 |
|  | GCF_027595525.1 |  | GCF_023195715.1 |
|  | GCF_028622135.1 |  | GCF_023375565.1 |
|  | GCF_030179805.1 |  | GCF_023375585.1 |
|  | GCF_030179835.1 |  | GCF_023375625.1 |
| *Enterobacter bugandensis* | GCF_004804375.1 |  | GCF_023650875.1 |
|  | GCF_004804395.1 |  | GCF_025021485.1 |
|  | GCF_015137655.1 |  | GCF_025999915.1 |
|  | GCF_019046905.1 |  | GCF_029536245.1 |
|  | GCF_020042625.1 |  | GCF_029542685.1 |
|  | GCF_020097235.1 |  | GCF_029542705.1 |
|  | GCF_020099235.1 |  | GCF_029542745.1 |
|  | GCF_020099255.1 |  | GCF_029718635.1 |
|  | GCF_023374275.1 |  | GCF_029834495.1 |
|  | GCF_900324475.1 |  | GCF_030179035.1 |
| *Enterobacter cancerogenus* | GCF_002850575.1 |  | GCF_030179635.1 |
|  | GCF_009648915.1 | *Enterobacter sichuanensis* | GCF_009036245.1 |
|  | GCF_019047785.1 | *Enterobacter soli* | GCF_000224675.1 |
|  | GCF_019665745.1 | *Enterobacter* sp. | GCF_000016325.1 |
|  | GCF_019880465.1 |  | GCF_000410515.1 |
|  | GCF_904425455.1 |  | GCF_000801755.2 |
| *Enterobacter chengduensis* | GCF_001984825.2 |  | GCF_001623605.1 |
|  | GCF_019273075.1 |  | GCF_001719105.1 |
|  | GCF_019394435.1 |  | GCF_001888805.2 |
| *Enterobacter cloacae* | GCF_000724505.1 |  | GCF_002787395.1 |
|  | GCF_000770155.1 |  | GCF_002863825.2 |
|  | GCF_000784865.1 |  | GCF_002952735.2 |
|  | GCF_000784905.1 |  | GCF_004006055.1 |
|  | GCF_001562175.1 |  | GCF_007035975.1 |
|  | GCF_001708345.1 |  | GCF_008271405.2 |
|  | GCF_002197345.1 |  | GCF_008931465.1 |
|  | GCF_002303275.1 |  | GCF_010692925.1 |
|  | GCF_002982195.1 |  | GCF_011765665.1 |
|  | GCF_003031445.1 |  | GCF_013375955.1 |
|  | GCF_003031755.1 |  | GCF_013615235.1 |
|  | GCF_003719615.1 |  | GCF_013749735.1 |
|  | GCF_004151605.1 |  | GCF_013782625.1 |
|  | GCF_004193715.1 |  | GCF_013784105.1 |
|  | GCF_004801515.1 |  | GCF_013927005.1 |
|  | GCF_004801535.1 |  | GCF_014298075.1 |
|  | GCF_004801595.1 |  | GCF_016082135.1 |
|  | GCF_009707405.1 |  | GCF_018074945.1 |
|  | GCF_012974405.1 |  | GCF_018075005.1 |
|  | GCF_013375935.1 |  | GCF_018075145.1 |
|  | GCF_013376815.1 |  | GCF_018075245.1 |
|  | GCF_013376835.1 |  | GCF_019378715.1 |
|  | GCF_014169295.1 |  | GCF_019378735.1 |
|  | GCF_014169635.1 |  | GCF_019378755.1 |
|  | GCF_014169815.1 |  | GCF_019378775.1 |
|  | GCF_015138375.1 |  | GCF_019378795.1 |
|  | GCF_016864075.1 |  | GCF_019378815.1 |
|  | GCF_016864095.1 |  | GCF_019378835.1 |
|  | GCF_016864115.1 |  | GCF_019443845.1 |
|  | GCF_018140965.1 |  | GCF_019502745.1 |
|  | GCF_019047105.1 |  | GCF_019880405.1 |
|  | GCF_019265065.1 |  | GCF_019968765.1 |
|  | GCF_019265085.1 |  | GCF_019968805.1 |
|  | GCF_019265105.1 |  | GCF_019968825.1 |
|  | GCF_019265125.1 |  | GCF_020971765.1 |
|  | GCF_019265145.1 |  | GCF_021725435.1 |
|  | GCF_019711235.1 |  | GCF_024734705.1 |
|  | GCF_020540925.1 |  | GCF_025152625.1 |
|  | GCF_020882135.1 |  | GCF_025502525.1 |
|  | GCF_021496745.1 |  | GCF_028735835.1 |
|  | GCF_021497605.1 | *Leclercia adecarboxylata* | GCF_001518835.1 |
|  | GCF_022220405.1 |  | GCF_004295325.1 |
|  | GCF_023238665.1 |  | GCF_006874705.1 |
|  | GCF_023920605.1 |  | GCF_008807335.1 |
|  | GCF_024917615.1 |  | GCF_009720165.1 |
|  | GCF_025426135.1 |  | GCF_011045715.1 |
|  | GCF_029873395.1 |  | GCF_011045735.1 |
|  | GCF_030166415.1 |  | GCF_011387015.1 |
|  | GCF_905232805.1 |  | GCF_020097395.1 |
|  | GCF_905322535.1 |  | GCF_020990425.1 |
|  | GCF_905331265.2 |  | GCF_023612295.1 |
|  | GCF_905331365.1 |  | GCF_023639785.1 |
| *Enterobacter cloacae* complex sp. | GCF_000783675.2 |  | GCF_025790845.1 |
|  | GCF_001922365.1 | *Leclercia pneumoniae* | GCF_017348915.1 |
|  | GCF_002055735.1 |  | GCF_018987305.1 |
|  | GCF_002192355.1 | *Leclercia* sp. | GCF_003336325.1 |
|  | GCF_002192395.1 |  | GCF_003336345.1 |
|  | GCF_002201815.1 |  | GCF_009734465.1 |
|  | GCF_002204775.1 |  | GCF_009734485.1 |
|  | GCF_002208095.1 |  | GCF_009740165.1 |
|  | GCF_002947755.1 |  | GCF_011290365.1 |
|  | GCF_002954165.1 |  | GCF_018513965.1 |
|  | GCF_003010695.1 |  | GCF_021117075.1 |
|  | GCF_003053755.1 | *Lelliottia amnigena* | GCF_001514515.1 |
|  | GCF_003071645.1 |  | GCF_001652505.2 |
|  | GCF_003204095.1 |  | GCF_002393405.1 |
|  | GCF_004355165.1 |  | GCF_002553545.1 |
|  | GCF_019056615.1 |  | GCF_003752235.2 |
|  | GCF_019056635.1 |  | GCF_004331765.1 |
|  | GCF_019056655.1 |  | GCF_013337605.1 |
|  | GCF_019856235.1 |  | GCF_016770935.1 |
|  | GCF_020149545.1 |  | GCF_016770955.1 |
|  | GCF_021165715.1 |  | GCF_016770975.1 |
|  | GCF_021869625.1 |  | GCF_016770995.1 |
|  | GCF_021869645.1 |  | GCF_016771075.1 |
|  | GCF_021869665.1 |  | GCF_019047465.1 |
|  | GCF_021869685.1 |  | GCF_019048185.1 |
|  | GCF_024599795.1 |  | GCF_019355955.1 |
|  | GCF_900322725.1 |  | GCF_021441185.1 |
| *Enterobacter cloacae* subsp. *cloacae* | GCF_000025565.1 |  | GCF_021498285.1 |
|  | GCF_023023085.1 |  | GCF_022352085.1 |
| *Enterobacter cloacae* subsp. *dissolvens* | GCF_000235765.1 |  | GCF_023970615.1 |
| *Enterobacter hormaechei* | GCF_000210775.1 |  | GCF_025641975.1 |
|  | GCF_001022015.1 |  | GCF_902160115.1 |
|  | GCF_001022055.1 |  | GCF_947072025.1 |
|  | GCF_001022075.1 | *Lelliottia aquatilis* | GCF_002922915.1 |
|  | GCF_001022255.1 |  | GCF_002923025.1 |
|  | GCF_001874505.1 |  | GCF_002923065.1 |
|  | GCF_002237465.1 |  | GCF_002923085.1 |
|  | GCF_003051945.2 |  | GCF_002923125.1 |
|  | GCF_003073995.1 |  | GCF_013337655.1 |
|  | GCF_003186415.1 |  | GCF_016771845.1 |
|  | GCF_003186565.1 | *Lelliottia jeotgali* | GCF_002271215.1 |
|  | GCF_003264955.1 | *Lelliottia nimipressuralis* | GCF_004115925.1 |
|  | GCF_003288475.1 |  | GCF_004402045.1 |
|  | GCF_003408555.1 |  | GCF_008244655.1 |
|  | GCF_003408575.1 |  | GCF_015319205.1 |
|  | GCF_003408595.1 |  | GCF_021083665.1 |
|  | GCF_003428425.1 | *Lelliottia steviae* | GCF_015209785.1 |
|  | GCF_003444755.1 | *Lelliottia* sp. | GCF_002869975.1 |
|  | GCF_003660125.1 |  | GCF_002870005.1 |
|  | GCF_003937295.2 |  | GCF_002870015.1 |
|  | GCF_003965345.2 |  | GCF_002922995.1 |
|  | GCF_004118875.1 |  | GCF_003051885.1 |
|  | GCF_005217135.2 |  | GCF_016859735.1 |
|  | GCF_005217155.2 |  | GCF_020532665.2 |
|  | GCF_008123985.1 |  | GCF_023278595.1 |
|  | GCF_008124025.1 |  | GCF_026672535.1 |
|  | GCF_008505035.1 |  | GCF_029967795.1 |
|  | GCF_008693905.1 |  | GCF_030234145.1 |
|  | GCF_008931325.1 |  | GCF_030234155.1 |
|  | GCF_008931405.1 |  | GCF_030234185.1 |
|  | GCF_008931525.1 |  | GCF_030238325.1 |
|  | GCF_008931585.1 |  | GCF_030238345.1 |
|  | GCF_008931645.1 |  | GCF_030238365.1 |
|  | GCF_008931785.1 |  | GCF_030238385.1 |
|  | GCF_009497055.1 |  | GCF_030238435.1 |
|  | GCF_009728975.1 |  | GCF_030238875.1 |

**Supplementary Table 2**. COG functional annotation of the predicted proteins in strain JS-SCA-14

| **COG** | **Functional category** | **Count** | **Ratio (%)** |
| --- | --- | --- | --- |
| A | RNA processing and modification | 1 | 0.0222 |
| B | Chromatin structure and dynamics | 0 | 0.0000 |
| C | Energy production and conversion | 217 | 4.8233 |
| D | Cell cycle control, cell division, chromosome partitioning | 39 | 0.8669 |
| E | Amino acid transport and metabolism | 361 | 8.0240 |
| F | Nucleotide transport and metabolism | 84 | 1.8671 |
| G | Carbohydrate transport and metabolism | 404 | 8.9798 |
| H | Coenzyme transport and metabolism | 127 | 2.8228 |
| I | Lipid transport and metabolism | 86 | 1.9115 |
| J | Translation, ribosomal structure and biogenesis | 181 | 4.0231 |
| K | Transcription | 321 | 7.1349 |
| L | Replication, recombination and repair | 187 | 4.1565 |
| M | Cell wall/membrane/envelope biogenesis | 267 | 5.9347 |
| N | Cell motility | 99 | 2.2005 |
| O | Posttranslational modification, protein turnover, chaperones | 145 | 3.2229 |
| P | Inorganic ion transport and metabolism | 292 | 6.4903 |
| Q | Secondary metabolites biosynthesis, transport and catabolism | 61 | 1.3559 |
| R | General function prediction only | 150 | 3.3341 |
| S | Function unknown | 1169 | 25.9836 |
| T | Signal transduction mechanisms | 161 | 3.5786 |
| U | Intracellular trafficking, secretion, and vesicular transport | 93 | 2.0671 |
| V | Defense mechanisms | 53 | 1.1780 |
| W | Extracellular structures | 1 | 0.0222 |
| Y | Nuclear structure | 0 | 0.0000 |
| Z | Cytoskeleton | 0 | 0.0000 |
| **Total** | | 4499 | **100** |

**Supplementary Table 3**. Hits of the 16S rRNA gene sequence of the strain JS-SCA-14 against EzBioCloud 16S rRNA gene sequence database

| **A^*^** | **B^*^** | **C^*^** | **D^*^** | **E^*^** | **F^*^** |
| --- | --- | --- | --- | --- | --- |
| 1 | *Lelliottia jeotgali* | PFL01 | KX709881 | 99.701715 | 91.723666 |
| 2 | *Buttiauxella izardii* | CCUG 35510 | QZWH01000019 | 99.383562 | 100 |
| 3 | *Lelliottia nimipressuralis* | LMG 10245 | Z96077 | 99.383139 | 100 |
| 4 | *Leclercia pneumoniae* | 4-9-1-25 | OK161083 | 99.341142 | 93.77565 |
| 5 | *Silvania hatchlandensis* | H19S6 | OM987253 | 99.181548 | 91.928865 |
| 6 | *Buttiauxella noackiae* | ATCC 51607 | LXEO01000077 | 99.178082 | 100 |
| 7 | *Buttiauxella warmboldiae* | CCUG 35512 | RPOH01000027 | 99.109589 | 100 |
| 8 | *Lelliottia amnigena* | NBRC 105700 | BCNN01000001 | 99.042408 | 100 |
| 9 | *Pseudomonas tritici* | SWRI145 | JABWQF010000088 | 99.042408 | 100 |
| 10 | *Enterobacter wuhouensis* | WCHEW120002 | SJOO01000031 | 98.974008 | 100 |
| 11 | *Leclercia tamurae* | H6S3 | OM987255 | 98.958333 | 91.928865 |
| 12 | *Leclercia adecarboxylata* | NBRC 102595 | BCNP01000062 | 98.905609 | 100 |
| 13 | *Buttiauxella gaviniae* | ATCC 51604 | LXEP01000074 | 98.90411 | 100 |
| 14 | *Silvania confinis* | H4N4 | OM987254 | 98.809524 | 91.928865 |
| 15 | *Huaxiibacter chinensis* | 155047 | OL712205 | 98.76881 | 100 |
| 16 | *Enterobacter huaxiensis* | 090008 | MK049964 | 98.75731 | 93.70725 |
| 17 | *Enterobacter kobei* | DSM 13645 | CP017181 | 98.70041 | 100 |
| 18 | *Enterobacter vonholyi* | E13 | VTUC01000023 | 98.69863 | 100 |
| 19 | *Citrobacter gillenii* | CDC 4693-86 | AF025367 | 98.632011 | 100 |
| 20 | *Enterobacter ludwigii* | EN-119 | JTLO01000001 | 98.632011 | 100 |
| 21 | *Scandinavium goeteborgense* | CCUG 66741 | MK558235 | 98.632011 | 100 |
| 22 | *Buttiauxella agrestis* | ATCC 33320 | JMPI01000079 | 98.630137 | 100 |
| 23 | *Buttiauxella ferragutiae* | ATCC 51602 | LXEQ01000068 | 98.630137 | 100 |
| 24 | *Kluyvera intermedia* | NBRC 102594 | BCYS01000084 | 98.562628 | 100 |
| 25 | *Citrobacter pasteurii* | CIP 55.13 | CDHL01000036 | 98.495212 | 100 |
| 26 | *Klebsiella aerogenes* | KCTC 2190 | CP002824 | 98.495212 | 100 |
| 27 | *Yokenella regensburgei* | ATCC 49455 | JMPS01000045 | 98.426813 | 100 |
| 28 | *Enterobacter cancerogenus* | ATCC 33241 | FYBA01000020 | 98.426813 | 100 |
| 29 | *Enterobacter chuandaensis* | 090028 | MK049966 | 98.426813 | 100 |
| 30 | *Enterobacter roggenkampii* | EN-117 | CP017184 | 98.424658 | 100 |
| 31 | *Raoultella ornithinolytica* | JCM 6096 | AJ251467 | 98.421414 | 100 |
| 32 | *Enterobacter sichuanensis* | WCHECl1597 | POVL01000141 | 98.358413 | 100 |
| 33 | *Citrobacter cronae* | Tue2_1 | MN548424 | 98.358413 | 100 |
| 34 | *Klebsiella spallanzanii* | SPARK_775_C1 | MN091365 | 98.358413 | 100 |
| 35 | *Buttiauxella brennerae* | DSM 9396 | AJ233401 | 98.356164 | 100 |
| 36 | *Enterobacter soli* | ATCC BAA-2102 | LXES01000062 | 98.356164 | 100 |
| 37 | *Enterobacter bugandensis* | EB-247 | FYBI01000003 | 98.290014 | 100 |
| 38 | *Kluyvera cryocrescens* | ATCC 33435 | AF310218 | 98.28938 | 96.032832 |
| 39 | *Dryocola boscaweniae* | H6W4 | OM971056 | 98.28869 | 91.928865 |

^*^A: Rank, B: Name, C: Strain, D: Accession, E: Pairwise Similarity (%), and F: Completeness (%).

**Supplementary Table 4**. List of top ten species with OrthoANI values when compared with the genome sequence of strain JS-SCA-14

| **Subject** | **Organism** | **Strain** | **OrthoANI**  **(%)** |
| --- | --- | --- | --- |
| GCF_002271215.1 | *Lelliottia jeotgali* | PFL01 | 91.3489 |
| GCF_016771845.1 | *Lelliottia aquatilis* | TZW17 | 91.1842 |
| GCF_013337655.1 | *Lelliottia aquatilis* | UMG3140 | 91.1708 |
| GCF_002922915.1 | *Lelliottia aquatilis* | 9827-07 | 91.1583 |
| GCF_002923065.1 | *Lelliottia aquatilis* | 6333-17 | 91.1519 |
| GCF_002923085.1 | *Lelliottia aquatilis* | 6332-17 | 91.1383 |
| GCF_002923125.1 | *Lelliottia aquatilis* | 6334-17 | 91.1242 |
| GCF_002923025.1 | *Lelliottia aquatilis* | 6331-17 | 91.0896 |
| GCF_021083665.1 | *Lelliottia nimipressuralis* | MEZLN61 | 90.7712 |
| GCF_902160115.1 | *Lelliottia amnigena* | 4928STDY7071390 | 90.6981 |

Results with unclassified genomes were not included in the table.

**Supplementary Table 5**. List of biosynthetic gene clusters predicted by antiSMASH

| **Region** | **Type** | **From** | **Most similar  known cluster** | **Similarity** | **PGPT-Pred^*^** |
| --- | --- | --- | --- | --- | --- |
| Region 1.1 | NRPS | 1,353,179-1,397,054 | amonabactin P 750 (NRP) | 57% | 78.95% (30 / 38) |
| Region 1.2 | thiopeptide | 1,705,306-1,731,607 | O-antigen (Saccharide) | 14% | 78.95% (15 / 19) |
| Region 1.3 | arylpolyene | 4,542,944-4,586,537 | aryl polyenes (Other) | 100% | 68.89% (31 / 45) |
| Region 1.4 | RiPP-like | 4,866,445-4,877,068 | - | - | 42.86% (3 / 7) |

*The ratio and number of genes that were annotated by PGPT-Pred for each biosynthetic gene cluster.

**Supplementary Table 6**. List of genomic islands predicted by IslandViewer^*^

| **Name** | **Chromosomal Location** | **Size** | **GI Prediction Method** |
| --- | --- | --- | --- |
| **IV4_01** | **23,957-33,543** | **9,586** | **IslandPath-DIMOB** |
| **IV4_02** | **25,756-32,769** | **7,013** | **SIGI-HMM** |
| IV4_03 | 379,886-383,934 | 4,048 | IslandPick |
| **IV4_04** | **386,604-391,542** | **4,938** | **IslandPick** |
| **IV4_05** | **391,688-397,235** | **5,547** | **IslandPick** |
| **IV4_06** | **629,213-640,747** | **11,534** | **IslandPath-DIMOB** |
| **IV4_07** | **971,703-986,128** | **14,425** | **SIGI-HMM** |
| **IV4_08** | **1,012,570-1,018,106** | **5,536** | **SIGI-HMM** |
| IV4_09 | 1,027,424-1,032,190 | 4,766 | IslandPick |
| **IV4_10** | **1,249,355-1,324,477** | **75,122** | **IslandPath-DIMOB** |
| **IV4_11** | **1,252,627-1,257,880** | **5,253** | **SIGI-HMM** |
| **IV4_12** | **1,285,181-1,291,741** | **6,560** | **IslandPick** |
| **IV4_13** | **1,286,737-1,291,356** | **4,619** | **SIGI-HMM** |
| **IV4_14** | **1,292,146-1,300,616** | **8,470** | **SIGI-HMM** |
| **IV4_15** | **1,300,843-1,326,938** | **26,095** | **IslandPick** |
| **IV4_16** | **1,945,748-1,965,372** | **19,624** | **IslandPath-DIMOB** |
| **IV4_17** | **1,947,461-1,954,230** | **6,769** | **IslandPick** |
| IV4_18 | 2,008,795-2,013,619 | 4,824 | IslandPick |
| **IV4_19** | **2,050,605-2,073,719** | **23,114** | **IslandPick** |
| **IV4_20** | **2,052,798-2,075,105** | **22,307** | **IslandPath-DIMOB** |
| **IV4_21** | **2,060,498-2,065,097** | **4,599** | **SIGI-HMM** |
| IV4_22 | 2,213,145-2,218,615 | 5,470 | IslandPick |
| **IV4_23** | **2,252,701-2,269,238** | **16,537** | **IslandPick** |
| **IV4_24** | **2,264,506-2,268,968** | **4,462** | **SIGI-HMM** |
| IV4_25 | 2,322,542-2,328,144 | 5,602 | IslandPick |
| IV4_26 | 2,335,715-2,341,255 | 5,540 | IslandPick |
| IV4_27 | 2,348,077-2,352,258 | 4,181 | IslandPick |
| **IV4_28** | **2,439,957-2,445,390** | **5,433** | **SIGI-HMM** |
| IV4_29 | 2,722,053-2,732,814 | 10,761 | IslandPick |
| **IV4_30** | **2,733,132-2,737,733** | **4,601** | **IslandPick** |
| **IV4_31** | **2,756,642-2,762,040** | **5,398** | **IslandPick** |
| **IV4_32** | **2,757,541-2,761,748** | **4,207** | **SIGI-HMM** |
| IV4_33 | 2,769,305-2,777,493 | 8,188 | IslandPick |
| IV4_34 | 2,779,229-2,784,123 | 4,894 | IslandPick |
| IV4_35 | 2,889,639-2,896,441 | 6,802 | IslandPick |
| **IV4_36** | **2,970,320-2,982,315** | **11,995** | **IslandPick** |
| **IV4_37** | **3,012,630-3,017,471** | **4,841** | **IslandPick** |
| **IV4_38** | **3,077,270-3,090,853** | **13,583** | **SIGI-HMM** |
| IV4_39 | 3,510,864-3,524,137 | 13,273 | IslandPick |
| **IV4_40** | **3,592,640-3,628,487** | **35,847** | **IslandPath-DIMOB** |
| **IV4_41** | **3,598,511-3,609,412** | **10,901** | **SIGI-HMM** |
| **IV4_42** | **3,612,984-3,619,570** | **6,586** | **SIGI-HMM** |
| **IV4_43** | **3,620,118-3,628,487** | **8,369** | **SIGI-HMM** |
| IV4_44 | 3,892,986-3,899,055 | 6,069 | IslandPick |
| IV4_45 | 3,902,166-3,906,503 | 4,337 | IslandPick |
| **IV4_46** | **3,906,554-3,925,471** | **18,917** | **IslandPick** |
| **IV4_47** | **3,908,116-3,926,854** | **18,738** | **IslandPath-DIMOB** |
| IV4_48 | 4,006,377-4,010,999 | 4,622 | IslandPick |
| **IV4_49** | **4,011,510-4,025,621** | **14,111** | **IslandPick** |
| **IV4_50** | **4,026,088-4,039,467** | **13,379** | **IslandPick** |
| **IV4_51** | **4,040,129-4,045,332** | **5,203** | **IslandPick** |
| **IV4_52** | **4,133,155-4,138,416** | **5,261** | **IslandPick** |
| **IV4_53** | **4,134,710-4,141,938** | **7,228** | **IslandPath-DIMOB** |
| **IV4_54** | **4,137,266-4,141,938** | **4,672** | **SIGI-HMM** |
| IV4_55 | 4,582,540-4,587,625 | 5,085 | IslandPick |

^*^Prediction results overlapping with those from Alien_Hunter were shown in bold face (see also Supplementary Table 7).

**Supplementary Table 7**. List of genomic islands predicted by Alien_Hunter^*^

| **Name** | **Chromosomal Location** | **Score^**^** | **Length (bp)** | **Name** | **Chromosomal Location** | **Score^**^** | **Length (bp)** |
| --- | --- | --- | --- | --- | --- | --- | --- |
| **GI_01** | **25,886-33,106** | **37.671** | **7,221** | GI_29 | 2,491,248-2,498,543 | 15.09 | 7,296 |
| GI_02 | 95,517-104,918 | 16.13 | 9,402 | GI_30 | 2,677,467-2,683,854 | 14 | 6,388 |
| GI_03 | 104,919-111,947 | 28.646 | 7,029 | **GI_31** | **2,736,492-2,748,732** | **24.971** | **12,241** |
| GI_04 | 126,315-133,407 | 20.248 | 7,093 | **GI_32** | **2,753,543-2,762,036** | **19.79** | **8,494** |
| GI_05 | 256,544-270,890 | 17.685 | 14,347 | **GI_33** | **2,982,139-2,988,259** | **19.577** | **6,121** |
| GI_06 | 287,133-294,176 | 21.958 | 7,044 | **GI_34** | **3,009,131-3,017,227** | **15.552** | **8,097** |
| GI_07 | 368,290-372,403 | 14.01 | 4,114 | **GI_35** | **3,077,237-3,090,933** | **71.009** | **13,697** |
| **GI_08** | **385,568-395,264** | **35.799** | **9,697** | GI_36 | 3,160,977-3,168,605 | 23.864 | 7,629 |
| GI_09 | 477,955-483,582 | 20.631 | 5,628 | GI_37 | 3,464,721-3,469,487 | 14.883 | 4,767 |
| **GI_10** | **630,918-640,969** | **56.562** | **10,052** | GI_38 | 3,564,058-3,571,311 | 22.49 | 7,254 |
| GI_11 | 922,443-933,005 | 24.151 | 10,563 | **GI_39** | **3,598,056-3,631,049** | **56.988** | **32,994** |
| **GI_12** | **970,924-987,087** | **30.588** | **16,164** | GI_40 | 3,669,056-3,680,163 | 19.752 | 11,108 |
| **GI_13** | **1,008,694-1,023,832** | **53.463** | **15,139** | **GI_41** | **3,914,985-3,926,116** | **20.554** | **11,132** |
| **GI_14** | **1,249,185-1,264,013** | **18.508** | **14,829** | **GI_42** | **4,018,420-4,024,203** | **15.637** | **5,784** |
| **GI_15** | **1,270,026-1,279,695** | **17.436** | **9,670** | **GI_43** | **4,034,194-4,045,349** | **23.678** | **11,156** |
| **GI_16** | **1,288,477-1,300,871** | **35.163** | **12,395** | **GI_44** | **4,131,779-4,142,300** | **27.539** | **10,522** |
| **GI_17** | **1,313,631-1,324,349** | **14.017** | **10,719** | GI_45 | 4,216,627-4,221,959 | 19.971 | 5,333 |
| GI_18 | 1,400,683-1,407,802 | 13.955 | 7,120 | GI_46 | 4,226,783-4,232,748 | 14.628 | 5,966 |
| GI_19 | 1,517,406-1,523,832 | 14.284 | 6,427 | GI_47 | 4,387,672-4,397,848 | 24.192 | 10,177 |
| GI_20 | 1,823,341-1,831,476 | 15.087 | 8,136 | GI_48 | 4,403,805-4,423,583 | 17.149 | 19,779 |
| GI_21 | 1,923,322-1,930,292 | 14.353 | 6,971 | GI_49 | 4,644,704-4,650,472 | 28.025 | 5,769 |
| **GI_22** | **1,947,404-1,957,996** | **34.064** | **10,593** | GI_50 | 4,737,732-4,743,639 | 24.064 | 5,908 |
| **GI_23** | **2,059,762-2,074,037** | **39.675** | **14,276** | GI_51 | 4,870,050-4,880,168 | 22.086 | 10,119 |
| GI_24 | 2,119,378-2,138,763 | 21.708 | 19,386 | GI_52 | 4,898,538-4,904,332 | 13.72 | 5,795 |
| GI_25 | 2,141,559-2,152,399 | 14.907 | 10,841 | GI_53 | 4,913,094-4,960,667 | 35.599 | 47,574 |
| GI_26 | 2,194,255-2,201,058 | 23.493 | 6,804 | GI_54 | 4,968,433-4,978,761 | 26.295 | 10,329 |
| **GI_27** | **2,252,652-2,269,211** | **19.241** | **16,560** | GI_55 | 4,988,309-4,996,994 | 18.819 | 8,686 |
| **GI_28** | **2,440,066-2,445,104** | **27.882** | **5,039** | GI_56 | 5,000,402-5,005,402 | 32.333 | 5,001 |

^*^Prediction results overlapping with those from IslandViewer were shown in bold face (see also Supplementary Table 6).
^**^Threshold: 13.439.

**Supplementary Table 8**. Comparative analysis of carbon source utilization by strains JS-SCA-14 and *Lelliottia jeotgali* PFL01

| **Carbon source** | **1^*^** | **2^*^** |
| --- | --- | --- |
| Glycerol | - | + |
| Erythritol | - | - |
| D-arabinose. | - | - |
| L-arabinose | + | + |
| D-ribose | + | + |
| D-xylose | + | + |
| L-xylose | - | - |
| D-xylose | - | - |
| Methyl-beta-D-xylopyranoside | - | - |
| D-galactose | + | + |
| D-glucose | + | + |
| D-fructose | + | + |
| D-mannose | + | + |
| L-sorbose | - | - |
| L-rhamnose | + | + |
| Dulcitol | - | - |
| Inositol | - | - |
| D-mannitol | + | + |
| D-sorbitol | - | + |
| Methyl-alpha-D-mannopyranoside | - | - |
| Methyl-alpha-D-glucopyranoside | - | - |
| N-acetylglucosamine | + | + |
| Amygdalin | - | - |
| Arbutin | - | - |
| Esculin ferric citrate | - | - |
| Salicin | - | - |
| D-cellobiose | + | + |
| D-maltose | + | + |
| D-lactose (bovine origin) | - | - |
| D-melibiose | + | + |
| D-saccharose (sucrose) | + | - |
| D-trehalose | + | + |
| Inulin | - | - |
| D-melezitose | - | - |
| D-raffinose | + | - |
| Amidon (starch) | - | - |
| Glycogen | - | - |
| Xylitol | - | - |
| Gentiobiose | + | - |
| D-turanose | - | - |
| D-lyxose | - | - |
| D-tagatose | - | - |
| D-fucose | - | - |
| L-fucose | - | - |
| D-arabitol | - | - |
| L-arabitol | - | - |
| Potassium gluconate | + | + |
| Potassium 2-ketogluconate | - | - |
| Potassium 5-ketogluconate | - | + |

^*^1: JS-SCA-14, 2: *Lelliottia jeotgali* PFL01.

**Supplementary Table 9**. Comparative analysis of enzyme activities in strains JS-SCA-14 and *Lelliottia jeotgali* PFL01

| **Enzyme** | **1^*^** | **2^*^** |
| --- | --- | --- |
| alkaline phosphatase | **+** | **+** |
| Esterase (C4) | **-** | **-** |
| Esterase Lipase (C8) | **-** | **-** |
| Lipase (C14) | **-** | **-** |
| Leucine arylamidase | **+** | **+** |
| Valine arylamidase | **+** | **-** |
| Cystine arylamidase | **+** | **+** |
| Trypsin | **-** | **-** |
| α-chymotrypsin | **-** | **-** |
| Acid phosphatase | **+** | **+** |
| Naphthol-AS-BI-phosphohydrolase | **+** | **+** |
| α-galatosidase | **+** | **-** |
| β-galactosidase | **+** | **+** |
| β-glucuronidase | **-** | **-** |
| α-glucosidase | **+** | **-** |
| β-glucosidase | **-** | **-** |
| N-acetyl-β-glucosaminidase | **-** | **-** |
| α-mannosidase | **-** | **-** |
| α-fucosidase | **-** | **-** |

^*^1: JS-SCA-14, 2: *Lelliottia jeotgali* PFL01.

**Supplementary Table 10**. Comparative analysis of cellular fatty acid composition in strains JS-SCA-14 and *Lelliottia jeotgali* PFL01

| **Fatty acid** | **1^*^** | **2^*^** |
| --- | --- | --- |
| Straight-chain saturated: |  |  |
| 12:0 | 2.98 | 4.33 |
| 13:0 | - | 0.82 |
| 14:0 | 7.37 | 7.74 |
| **16:0** | **26.18** | **22.97** |
| 17:0 | 0.78 | 2.15 |
| 18:0 | 0.25 | - |
| Unsaturated: |  |  |
| 17:0 cyclo | 9.76 | 9.99 |
| 17:1 ω8c | - | 0.82 |
| 19:0 cyclo ω8c | 1.38 | 1.09 |
| Summed features: |  |  |
| 2 | 9.97 | **11.56** |
| **3** | **22.49** | **16.04** |
| **8** | **18.83** | **22.49** |

^*^1: JS-SCA-14, 2: *Lelliottia jeotgali* PFL01. -, Not detected. Summed features represent two or three fatty acids that were inseparable by the MIDI system. Summed feature 2, C12 : 0 aldehyde (unknown); 3, C16 : 1ω7c/C16 : 1ω6c; 8, C18 : 1ω7c/C18 : 1ω6c. Composition percentages higher than 10 % are indicated in bold.
